# Cerdioxid: MAK-Begründung

**DOI:** 10.34865/mb130638d9_4or

**Published:** 2024-12-23

**Authors:** Andrea Hartwig, MAK Commission

**Affiliations:** 1 Institut für Angewandte Biowissenschaften. Abteilung Lebensmittelchemie und Toxikologie. Karlsruher Institut für Technologie (KIT) Adenauerring 20a, Geb. 50.41 76131 Karlsruhe Deutschland; 2 Ständige Senatskommission zur Prüfung gesundheitsschädlicher Arbeitsstoffe. Deutsche Forschungsgemeinschaft, Kennedyallee 40, 53175 Bonn, Deutschland. Weitere Informationen: Ständige Senatskommission zur Prüfung gesundheitsschädlicher Arbeitsstoffe | DFG

**Keywords:** Cerdioxid, Lunge, Entzündung, Fibrose, humanäquivalente Konzentration, MAK-Wert, maximale Arbeitsplatzkonzentration, Spitzenbegrenzung

## Abstract

The German Senate Commission for the Investigation of Health Hazards of Chemical Compounds in the Work Area (MAK Commission) summarized and evaluated the data for cerium dioxide [1306-38-3] to derive an occupational exposure limit value (maximum concentration at the workplace, MAK value) considering all toxicological end points. Relevant studies were identified from a literature search and also unpublished study reports were used. The critical effects are inflammation and fibrosis in the lungs of humans and rats. With decreasing particle size, the ratio of the two stable cerium valence states Ce^3+^/Ce^4+^ increases in favour of Ce^3+^. This leads to altered physico-chemical properties, including an increased redox potential (Ce^3+^/Ce^4+^) and higher solubility. In a biological system, the toxicity of cerium dioxide particles is primarily due to their redox potential at the particle surface. However, a toxic effect due to partly solubilized cerium dioxide cannot be excluded. In a long-term inhalation study with nanoscale cerium dioxide particles (NM-212), cerium phosphate and cerium oxide were clearly detected in rat bone tissue but it remains unknown in which form cerium dioxide enters the bones and is incorporated there as cerium phosphate. In a 2-year inhalation study in rats, using nanoscale cerium dioxide particles with a more extensive investigation of the lung, granulomatous inflammation and interstitial fibrosis in the lung occurred at the lowest concentration tested of 0.1 mg/m^3^ and above. Therefore, a NOAEC cannot be established and 0.1 mg/m^3^ of cerium dioxide is regarded as LOAEC. Corresponding to the LOAEC, a human equivalent concentration of 11.2 μg/m^3^ was calculated and a MAK value of 2 μg cerium dioxide/m^3^ has been set for the respirable fraction. Additionally, the substance has been classified in Peak Limitation Category II with an excursion factor of 8. In the absence of a prenatal developmental toxicity study which encompasses the entire organogenesis and includes complete teratogenicity testing, cerium dioxide has been assigned to Pregnancy Risk Group D. Carcinogenic effects in humans cannot be derived for cerium dioxide. The NOAEL for clastogenic effects of cerium dioxide in rats was 30 mg/kg body weight and day. After applying toxicokinetic scaling, this corresponds to an air concentration of 5.26 mg/m^3^. As the margin to the MAK value is sufficiently large, cerium dioxide has been assigned to Germ Cell Mutagen Category 5. Cerium dioxide is not absorbed through the skin in toxicologically relevant amounts. Available data demonstrate no sensitizing potential.

**Table:** 

**MAK-Wert (2023)**	**0,002 mg/m^3^ A**
**Spitzenbegrenzung (2023)**	**Kategorie II, Überschreitungsfaktor 8**
	
**Hautresorption**	**–**
**Sensibilisierende Wirkung**	**–**
**Krebserzeugende Wirkung**	**–**
**Fruchtschädigende Wirkung (2023)**	**Gruppe D**
**Keimzellmutagene Wirkung (2023)**	**Kategorie 5**
	
**BAT-Wert**	**–**
	
Synonyma	Cer(IV)-oxid
Chemische Bezeichnung (IUPAC-Name)	Dioxocer
CAS-Nr.	1306-38-3
Formel	CeO_2_
Molmasse	172,12 g/mol (IFA 2023)
Schmelzpunkt	> 400 °C bei 1013 hPa (ECHA 2022)
Dichte	7,3 g/cm^3^ bei 20 °C (IFA 2023) 7,2 g/cm^3^ bei 20,3 °C (ECHA 2022)
Dampfdruck bei 20 °C	–
log K_OW_	–
Löslichkeit	< 0,123 µg/l Wasser bei 20 °C, pH-Wert 6 (ECHA 2022)
Härte (nach Mohs)	6**–**7 (Belkhir et al. 2009)
Kristallsystem/Raumgruppe	Kubisch/Fm3m (Morris et al. 1984)

Zitierte unveröffentlichte toxikologische Studien von Firmen wurden der Kommission zur Verfügung gestellt.

Cer (auch Cerium genannt) gehört zur Gruppe der Lanthanoide. Es ist das häufigste Seltene-Erde-Element (ca. 50 mg/kg) in der Erdkruste (National Minerals Information Center 2004). In der Natur ist Cer zusammen mit anderen Lanthanoiden in den Mineralien Alanit, Bastanit, Monazit, Cerit und Samarskit zu finden, wobei nur Bastanit und Monazit von kommerziellem Interesse sind (Haynes et al. 2014; Kilbourn 2011). Aufgrund seiner unter den Lanthanoiden einzigartigen Stabilität im vierwertigen Zustand kann Cer von den anderen Lanthanoiden über Oxidation und nachfolgende Löslichkeitsfiltration getrennt werden (Reinhardt und Winkler 2012). Elementares Cer ist sehr reaktiv und wird sehr leicht oxidiert. In Verbindungen existiert Cer im dreiwertigen (Ce(III)) oder vierwertigen (Ce(IV)) Zustand (Kilbourn 2011; Reinhardt und Winkler 2012). Cer(IV)-oxid (Cerdioxid, CeO_2_) ist die am häufigsten vorkommende Verbindung des Cers. In reiner Form ist Cerdioxid cremefarben bis hellgelb. Kommerzielle Produkte, die für Schleif- und Polierzwecke eingesetzt werden, haben durch enthaltene Verunreinigungen (z. B. Neodym, Praseodym) meist braune Farbtöne (US EPA 2009). Neben Cerdioxid existiert auch Cer(III)-oxid (Ce_2_O_3_), welches farblose Kristalle bildet (Binnewies et al. 2016).

Expositionen gegen kommerziell verwendete Cer-Verbindungen erfolgen meist in Form von Cerdioxid. Es wird entweder in Reinform oder konzentriert als Poliermittel für Glasspiegel, Glasplatten, Fernsehröhren, optische Linsen sowie in der Präzisionsoptik eingesetzt (Kilbourn 2011; Reinhardt und Winkler 2012). Cerdioxid wird auch als Glasbestandteil gegen Solarisation und Entfärbung verwendet (Reinhardt und Winkler 2012). Zudem findet Cerdioxid Verwendung in Abgasreinigungssystemen von Benzinmotoren und in Dieselfahrzeugkatalysatoren zur Reduktion der Partikelemission (HEI 2001; Reinhardt und Winkler 2012). 

## 1 Allgemeiner Wirkungscharakter

Cerdioxid-Partikel akkumulieren mit einer sehr hohen Biopersistenz in der Lunge und im lymphoretikulären System des Menschen. Durch das Einatmen von Cer-haltigen Stäuben können bei exponierten Beschäftigten individuell stark variierende Lungenschädigungen wie Pneumokoniosen ausgelöst werden.

In einer 2-Jahre-Inhalationsstudie mit nanoskaligen Cerdioxid-Partikeln an Ratten ruft bereits eine Expositions­konzentration von 0,1 mg/m^3^ eine statistisch signifikant erhöhte granulomatöse Entzündung sowie eine interstitielle Fibrose der Lunge hervor. Das inhalierte nanoskalige Cerdioxid erreicht zudem das Knochengewebe, wo es in Form von Agglomeraten oder Cerphosphat gespeichert wird. 

Nach 28-tägiger oraler Gabe an Ratten treten ab 300 mg/kg KG Aktivitätssteigerungen der alkalischen Phosphatase und der Laktatdehydrogenase im Blut auf.

Eine dreimalige intratracheale Gabe von 0,1 mg/Tier während der Trächtigkeit von Mäusen führt bei den Nachkommen zur Beeinträchtigung der Alveolen-Bildung in der Lunge und geringeren Körpergewichten.

In Fütterungsstudien an Ratten treten Mikronuklei in Leukozyten nach Gabe von 300 mg nanoskaligem Cerdioxid/kg KG und Tag auf.

In der 2-Jahre-Studie ist Cerdioxid bis zur höchsten getesteten Konzentration von 3 mg/m^3^ nicht kanzerogen an Ratten.

Es liegen keine Hinweise auf eine sensibilisierende Wirkung von Cerdioxid vor.

## 2 Wirkungsmechanismus

Mikro- wie auch nanoskaliges Cerdioxid weist eine spezifische Toxizität auf. Mit abnehmender Partikelgröße erhöht sich der Anteil der beiden stabilen Cer-Valenzzustände zugunsten des Ce^3+^-Anteils sowie die Löslichkeit der Partikel. Bedingt durch die Defektstruktur des Cerdioxids ist eine vollständige Oxidation zu Ce^4+^ an der Partikeloberfläche nicht möglich, so dass immer ein Ce^3+^-Anteil nachgewiesen werden kann. Cerdioxid-Partikel wirken daher im biologischen System sowohl aufgrund der katalytischen Fähigkeit (Ce^3+^/Ce^4+^-Redoxpotential) an der Partikeloberfläche, als auch aufgrund ihrer löslichen Spezies toxisch (Dekkers et al. 2017; Özkan et al. 2020). 

Bei Betrachtung der histopathologischen Befunde zeigen sich granulomatöse Veränderungen mit einem Lympho­zytensaum (sog. Immungranulome), welche auf eine immunologische Reaktion hinweisen (Ernst et al. 2018; Ma 2020).

Bei beruflich exponierten Personen konnte Cer noch Jahrzehnte nach Expositionsende in der Lunge nachgewiesen werden. Neben der hohen Biopersistenz von Cerdioxid-Partikeln in der Lunge deuten inhalative Tierstudien zusätzlich auf eine zytotoxische Wirkung und auf eine Löslichkeit von Cerdioxid in der Lunge hin. So konnten in einer Inhalationsstudie mit nanoskaligen Cerdioxid-Partikeln (NM-212) eindeutig Cerdioxid-Agglomerate und Cerphosphat im Knochengewebe von Ratten nachgewiesen werden (Tentschert et al. 2020).

Aus den vorliegenden humanmedizinischen Fallberichten geht hervor, dass eine inhalative Exposition mit Cer-haltigen Stäuben zu individuell stark variierenden Schädigungen der Lunge führen kann. Zum einen können stark erhöhte Lanthanoid-Konzentrationen im Lungengewebe oder in der Bronchialflüssigkeit nachgewiesen werden, ohne dass klinische Symptome zu beobachten sind und zum anderen werden interstitielle Cer-Pneumokoniosen unterschiedlicher Ausprägung bis hin zu Emphysem und interstitieller Fibrose diagnostiziert (siehe Abschnitt 4.1 und 4.2). Weitergehende Humandaten oder epidemiologische Studien liegen nicht vor. Adverse Effekte, wie eine alveoläre/interstitielle Infiltration mit Entzündungszellen, eine multifokale alveoläre/interstitielle granulomatöse Entzündung und eine geringgradige interstitielle Fibrose, wenn auch nicht statistisch signifikant, wurden auch im Tierversuch nach inhalativer Expositionskonzentration von bereits 0,1 mg Cerdioxid/m^3^ beobachtet. Auffällig war auch hier die individuelle Variabilität der Stärke der Befunde (siehe Abschnitt 5.1 und 5.2).

In-vivo-Studien untermauern die Hinweise auf eine proinflammatorische Wirkung des Cerdioxids. NM-212-Cerdioxid-Nanopartikel wurden in Konzentrationen von 0; 0,1; 0,3; 1 und 3 mg/m^3^ bei weiblichen Wistar-Ratten inhalativ eingesetzt und die Expression von 391 Genen in den alveolären Typ II-Zellen (AEII-Zellen), Leber und Niere nach einer ein-, 28- oder 90-tägigen Exposition untersucht. Die Auswertung der verwendeten PCR-Arrays ergab insgesamt 30 regulierte Gene aus den Bereichen „Entzündung, oxidativer Stress, DNA-Reparatur, Apoptose und Lungenkrebs“. Mit dem Anstieg der Expositionsdauer und der Cerdioxid-Konzentration stieg auch die Anzahl der verändert exprimierten Gene an. Von allen Entzündungsmediatoren waren die Chemokine Ccl2, Ccl7, Ccl17 und Ccl22 am deutlichsten hochreguliert. Die stärkste Expression wurde nach 90 Tagen Exposition bei einer Konzentration von 3,0 mg/m^3^ beobachtet. Die Entzündungsmediatoren Ccl3, Ccl4, Ccl24, Il-1α, Il-1β und Il-1rn waren bereits bei niedrigen Konzentrationen (0,1; 0,3 mg/m^3^) mit ähnlichen Expressionsmustern hochreguliert. Die deutlichsten Effekte waren auch hier nach einer Exposition von 90 Tagen zu sehen. Eine zeit- und konzentrationsabhängige Steigerung der Expression konnte ebenfalls für die Gene Lpo und Noxo1 aus dem Bereich „oxidativer Stress“ aufgezeigt werden. Die immunhistochemische Untersuchung der Lunge nach einer 90-tägigen Exposition gegen 3,0 mg/m^3^ der Cerdioxid-Nanopartikel ergab ­zudem eine erhöhte Proteinexpression von Lpo. Auch in Leber und Niere waren hauptsächlich Gene aus dem Bereich „Entzündung“ und „oxidativer Stress“ verändert, jedoch auch DNA-Reparatur-Gene (Schwotzer et al. 2018).

Auch in vitro induzierten nano- und mikroskalige Cerdioxid-Partikel (Nano-Ce: 0,009–0,015 µm; Oberfläche 71,3 m^2^/g, Mikro-Ce: 5 µm, Oberfläche 0,6 m^2^/g) Entzündungseffekte in Lungenzellen. In einer Konzentration von 53 µg/cm^2^ oder 100 µl/ml führten sie zu einem geringen Anstieg des inflammatorischen Markers IL-6 in Beas-2B-Zellen (immortalisierte humane Bronchial-Epithelzellen) und NHBE-Zellen (primäre humane Bronchial-Epithelzellen) (Veranth et al. 2007).

Die Zytotoxizität von Cerdioxid wurde in einer In-vitro-Studie mit einer Expositionszeit von 20 Stunden an pulmonalen Alveolarmakrophagen aus Sprague-Dawley-Ratten überprüft. Cerdioxid zeigte sich bei der Untersuchung der Zellviabilität als gering toxisch mit mindestens 80 % Viabilität bis 1000 µM. Cerdioxid beeinflusste die Freisetzung von lysosomalen Enzymen (saure Phosphatase, Cathepsin D) nicht. Die morphologische Untersuchung mittels Transmissionselektronenmikroskopie (TEM) ergab eine Abnahme der Zellen, deren Morphologie im Einklang mit der Kontrollpopulation steht, bei einer laut Autoren geringen Zunahme von Zellen mit einer strukturlosen Oberfläche (Palmer et al. 1987).

## 3 Toxikokinetik und Metabolismus

### 3.1 Aufnahme, Verteilung, Ausscheidung

Lanthanoide verhalten sich in biologischen Systemen als Calciumanaloga, was bedeutet, dass dreiwertige Lanthanoide Calciumionen an Metallbindungsstellen ersetzen können (Carrillo-López et al. 2010).

Die Lanthanoid-Verbindungen lassen sich in un- oder schwerlösliche (Oxide, Carbonate), lösliche (Chloride, Nitrate, Acetate) und Chelat-Verbindungen unterteilen.

Cerdioxid weist eine sehr geringe Wasserlöslichkeit auf (< 0,123 µg/l bei 20 °C und pH-Wert 6,01–6,41) und zählt daher zu den schwer löslichen Cer-Verbindungen. Der größte Teil der verfügbaren Information zur Resorption von Lanthanoid-Verbindungen liegt zu den löslichen Lanthanoiden vor. Die verschiedenen Lanthanoid-Verbindungen zeigen unterschiedliche Organverteilungen und Exkretionsraten. Für Lanthan-Oxide wurde in In-vitro-Studien eine gastrointestinale Bioverfügbarkeit von ungefähr 6 % nachgewiesen (Lambert et al. 1993).

Ein Fallbericht betrifft einen Patienten (58 Jahre bei der Erstvorstellung), der zuvor beruflich (Schleifer und Filmvorführer) gegen Seltene-Erden-Elemente und Asbest exponiert war. Bei der Untersuchung ergaben sich keine Hinweise auf eine Retention von Asbest. Cer, Lanthan und phosphathaltige Partikel wurden hingegen in Alveolarmakrophagen aus der bronchoalveolären Lavage nachgewiesen. 70 % der Partikel aus dem Lungengewebe enthielten Cer. Cer sowie phosphathaltige Partikel zeigten sich zudem in interstitiellen Makrophagen und in elastischen Fasern des Lungengewebes. Da der letzte Kontakt des Patienten mit Seltene-Erden-Metallen 15 Jahre zurücklag, sollten diese Elemente laut Autoren als biopersistent betrachtet werden (Pairon et al. 1995). 

In einer retrospektiven Studie wurden bronchoalveoläre Lavageflüssigkeit und Lungengewebe auf Cer-haltige Partikel untersucht. Bei sieben Patienten wurde eine hohe Retention (mindestens 5-fach im Vergleich zur Kontrolle) von Cer-haltigen Partikeln nachgewiesen. Die Zeitspanne seit der letzten Exposition lag bei diesen Patienten zwischen null und 29 Jahren (Pairon et al. 1994). 

Untersuchungen zur Verteilung von Cer-Verbindungen im Körper lassen darauf schließen, dass resorbiertes Cerdioxid vor allem in der Leber sowie in geringerem Maße in den Knochen abgelagert wird und dort über sehr lange Zeiträume akkumuliert (IFA 2023, Stockinger 1993).

Kolloidale Cer-Verbindungen können auch in den Lymphknoten deponiert werden. Die Ausscheidung ist mit der Gallenflüssigkeit, evtl. auch nach einer direkten Passage durch die Darmwände mit den Faeces, möglich. Mit einer Elimination über die Niere ist nur in einem geringen Maße zu rechnen. Die Ausscheidung unmittelbar nach stattgefundener Exposition ist insgesamt gering (IFA 2023).

Systemisch verfügbare Cer-Verbindungen scheinen vorrangig über die Faeces eliminiert zu werden. Nur ein geringer Anteil (< 10 %) wird mit dem Harn ausgeschieden (Durbin et al. 1956). 

#### 3.1.1 Inhalation

Fallberichte und retrospektive berufsbezogene Untersuchungen liefern Hinweise auf eine begrenzte Resorption von Cer-Partikeln in der Lunge nach inhalativer Exposition (US EPA 2009) (siehe Abschnitt 4.2). Wie andere schwerlösliche Partikel verhält sich auch Cerdioxid entsprechend seiner aerodynamischen Eigenschaften bei der Ablagerung innerhalb des Respirationstraktes (Schulz et al. 2000). 

Cer-Verbindungen werden grundsätzlich anhand ihrer erwarteten Clearance-Rate aus dem humanen Respirationstrakt in drei Kategorien unterteilt. Schwerlösliche Oxide und Hydroxide von Cer gehören zur Kategorie mit langsamer Clearance (Jahre). Nur ein Prozent der abgelagerten Partikel werden vermutlich aus dem Respirationstrakt resorbiert. Nitrate, Phosphate und Chloride des Cers gehören zur Kategorie mit mittlerer Clearance (Wochen). Wahrscheinlich werden 5–10 % der abgelagerten Partikel aus den nasopharyngealen und 50 % aus den tracheobronchialen Regionen ins Blut resorbiert. Die leichter löslichen Sulfate und Sulfite des Cers werden schneller ausgeschieden (HEI 2001). 

Wie in einem biokinetischen Modell für ^144^Ce in Alumosilikatpartikeln, die von Beagle-Hunden eingeatmet wurden, gezeigt, kommt es wie mit anderen schwerlöslichen Partikeln initial zu einer schnellen Elimination über die mukoziliäre Clearance und nachfolgend zu einem möglichen Abschlucken des Materials (Boecker und Cuddihy 1974; Shyr et al. 1991). 

Die Verteilung, Akkumulation und Elimination von Cerdioxid unterschiedlicher primärer Partikelgrößen (< 5000; 40; 5–10 nm) wurde in einer Studie an männlichen Wistar-Ratten verglichen. Jeweils drei Ratten pro Gruppe wurden einmalig oder wiederholt (max. 20 ×) an sechs Stunden pro Tag gegen Cerdioxid-Partikel nur über die Nase exponiert. Die verwendeten Partikel wiesen eine unterschiedliche Primärpartikelgröße auf, ähnelten sich jedoch in ihrem massenmedianen aerodynamischen Durchmesser (MMAD). Die charakteristischen Eigenschaften und die eingesetzten Konzentrationen der verwendeten Partikel sind in Tabelle 1 dargestellt (Geraets et al. 2012).

**Tab.1:** Eigenschaften der verwendeten Cerdioxid-Partikel in einer Studie an Wistar-Ratten (Geraets et al. 2012)

	**NM-213**	**NM-212**	**NM-211**
Nominale Primärpartikelgröße (nm)	< 5000	40	5–10
Mittlere Primärpartikelgröße (100 Partikel), gemessen mit TEM (Feret-Durchmesser, nm)	Probe zu groß für TEM Messung (> 500 nm)	27,3 ± 13,6 (22,4 ± 10,9)	13,0 ± 3,2 (10,7 ± 2,6)
Äquivalenter Kreisdurchmesser (nm)	Kleinste beobachtete Größe: 9 (6)		
Primäre Partikelgröße, gemessen mit SEM (Feret-Durchmesser, nm)	615,3 ± 430,5	28,4 ± 10,4	44,9 ± 14,6
MMAD mit aerodynamischem Partikelmessgerät (µm)	1,40 ± 0,11 (1,64)^a)^	1,17 ± 0,34 (2,07)^a)^	1,02 ± 0,04 (1,82)^a)^
CMD mit Scanning Mobility Particle Sizer (µm)	0,34 ± 0,06 (1,52)^a)^	0,25 ± 0,07 (1,78)^a)^	0,21 ± 0,03 (1,76)^a)^
BET-Oberfläche (m^2^/g)	3,73 ± 0,01	27,15 ± 0,19	63,95 ± 0,30
Durchmesser (d) aus BET-Oberfläche (nm)^b)^	213	29	12
Anzahlkonzentration (10^6^ Partikel/cm^3^)	0,68 ± 0,46	1,1 ± 0,70	1,79 ± 0,42
Massenkonzentration (mg/m^3^)	55,00 ± 6,20	19,95 ± 13,21	10,79 ± 0,82
Geschätzte inhalierte Gesamtdosis (mg)^c)^	4,24	1,54	0,83

Mittelwerte ± Standardabweichung

BET: Bestimmung der spezifischen Oberfläche mittels Brunauer-Emmett-Teller-Methode; CMD: Count Median Diameter (Partikelzahl-medianer Durchmesser); MMAD: massenmedianer aerodynamischer Durchmesser; SEM: Rasterelektronenmikroskopie (Scanning Electron Microscopy); TEM: Transmissionselektronenmikroskopie;

a) geometrische Standardabweichung

b) berechnet nach der Formel Durchmesser = 6/(Fläche × Dichte)

c) berechnet nach der Formel: Atemzugvolumen × Atemfrequenz × Expositionskonzentration × Expositionsdauer (analog zu den Parametereinstellungen des MPPD-Modells; das Atemzugvolumen betrug 2,10 ml und die Atemfrequenz 102/min)

Bereits nach einmaliger Exposition konnten Cer-Partikel in allen untersuchten Organen nachgewiesen werden, wobei sich der größte Anteil davon in der Lunge befand. Von den mikroskaligen Cerdioxid-Partikeln (NM-213, < 5000 nm) wurden 12,2 % des inhalierten Cerdioxids in der Lunge nachgewiesen. Für die beiden nanoskaligen Partikel lagen die Werte bei 5,6 % (NM-212, 40 nm) und 9,4 % (NM-211, 5–10 nm). Die Verteilung innerhalb des Atemtrakts ist in Tabelle 2 dargestellt und ähnelte sich bei allen drei getesteten Cerdioxid-Partikeln.

**Tab.2:** Partikeldeposition (Fraktion) von Cerdioxid innerhalb des respiratorischen Systems von Wistar-Ratten auf Grundlage der Schätzungen des MPPD-Depositionsmodells (Geraets et al. 2012)

**Region des Respirationstrakts**	**NM-213**			**NM-212**			**NM-211**		
	**MMAD**	**CMD**	**Primäre Partikelgröße**	**MMAD**	**CMD**	**Primäre Partikelgröße**	**MMAD**	**CMD**	**Primäre Partikelgröße**
Kopf	0,45	0,51	0,67	0,40	0,52	0,19	0,33	0,47	0,13
Tracheobronchial	0,03	0,03	0,04	0,03	0,02	0,10	0,03	0,02	0,07
Pulmonal	0,07	0,06	0,07	0,08	0,05	0,31	0,09	0,06	0,29
Insgesamt	0,54	0,59	0,78	0,50	0,59	0,60	0,44	0,55	0,49

Standardeinstellungen für Ratte: forcierte Atmungskapazität: 4 ml, Kopfvolumen: 0,42 ml, Nasenatmung, Atemzugvolumen: 2,1 ml, Atemfrequenz: 102/min, Inspirationsfraktion: 0,5, keine Pause, Dichte der Cerdioxid-Partikel: 7,65 g/cm^3^

CMD: Count Median Diameter (Partikelzahl-medianer Durchmesser); MMAD: massenmedianer aerodynamischer Durchmesser

Der Anteil des inhalierten Cerdioxids im extrapulmonalen Gewebe lag für alle untersuchten Partikel unter 0,2 %. Nach einmaliger Exposition gegen mikroskalige Cerdioxid-Partikel (NM-213, < 5000 nm) nahmen die Konzentrationen im extrapulmonalen Gewebe in folgender Reihung ab: Epididymis > Gehirn > Milz > Leber > Niere > Hoden. Nach wiederholter Exposition wurde der Unterschied bei der Verteilung zwischen den einzelnen Organen zunehmend geringer. Im Blut war in dieser Studie kein Cer nachweisbar. Die Autoren geben als mögliche Aufnahmewege in die extrapulmonalen Gewebe das lymphatische oder Kreislaufsystem oder den mukoziliären Transport mit nachfolgender oraler Exposition an. Zudem ist auch ein Aufnahmeweg über die olfaktorischen Nerven denkbar. Die wiederholte Exposition gegen alle getesteten Cerdioxid-Partikel führte zu einer statistisch signifikanten Zunahme der Cerdioxid-Konzentration in den Geweben, was auf eine Akkumulation hindeutet. Die Cerdioxid-Partikel wurden nur sehr langsam aus dem Gewebe freigesetzt, nach 72 Stunden war nur ein sehr geringer Anteil eliminiert worden (Geraets et al. 2012). In dieser Studie wurden die Cer-Gehalte mit dem Aufschlussverfahren mittels ICP-AES bzw. ICP-MS untersucht. Obwohl diese Methoden keine Spezieszuordnung zulassen, wurden in der Publikation die ermittelten Cer-Gehalte Cerdioxid gleichgesetzt. Durchgeführte Löslichkeitsversuche, zu denen keine näheren Angaben vorliegen, ergaben eine nur sehr geringe Löslichkeit von Cerdioxid. Daher schließen die Autoren die Bildung von Cer-Ionen als den der Verteilung zugrundeliegenden Mechanismus aus. Neuere Studien mit in Wasser schwer löslichen Nanopartikeln zeigen jedoch, dass es im Körper zu Lösungsvorgängen kommen kann, die hinsichtlich der Wasserlöslichkeit nicht zu vermuten gewesen wären. So weisen z. B. in Wasser schwer lösliche Bariumsulfat-Nanopartikel (NM-220) eine sehr kurze Retentionszeit auf und sind nach intratrachealer Installation bei Ratten im Knochen mittels Plasma-Massenspektrometrie (ICP-MS) nachweisbar (Keller et al. 2020; Molina et al. 2019).

In einer 2-Jahre-Inhalationsstudie gemäß OECD-Prüfrichtlinie 453 wurde die Verteilung von nanoskaligen Cerdioxid-Partikeln (NM-212) im Körper weiblicher Wistar-Ratten untersucht. An fünf Tagen pro Woche für sechs Stunden pro Tag wurde eine Ganzkörperexposition gegen Cerdioxid-Aerosolkonzentrationen von 0; 0,1; 0,3; 1 oder 3 mg/m^3^ durchgeführt. Die Tiere wurden nach drei, zwölf (jeweils n = 3) oder nach 24 Monaten (jeweils n = 4) untersucht. Die verwendeten Cerdioxid-Nanopartikel mit der Bezeichnung NM-212 wiesen eine primäre Partikelgröße von 28,4 nm, eine Oberfläche von 27,2 m^2^/g (BET), eine Wasserlöslichkeit von < 1 µg/l und eine Reinheit von > 99,5 % auf. Die Größenverteilung der Partikel im Aerosol ergab mikroskalige Agglomerate (1,4–2,3 µm) und variierte leicht zwischen den Gruppen (siehe Tabelle 3) (Tentschert et al. 2020).

**Tab.3:** Gemessene Expositionskonzentrationen und Größenverteilung der Cerdioxid-Partikel in einer Studie an Wistar-Ratten (Tentschert et al. 2020)

**Zielkonzentration ** **[mg/m³]**	**Gemessene Konzentration** **[mg/m³ ± SD]**	MMAD [μm] / GSD
0,1	0,1 ± 0,1	2,3/2,4
0,3	0,3 ± 0,1	1,7/2,3
1,0	1,0 ± 0,1	1,5/2,3
3,0	3,0 ± 0,4	1,4/2,1

GSD: geometrische Standardabweichung; MMAD: massenmedianer aerodynamischer Durchmesser; SD: Standardabweichung

Die Cer-Konzentration und -Verteilung in den einzelnen Organen wurde mit Techniken wie ICP-MS (Massenspektro­metrie mit induktiv gekoppeltem Plasma), ToF-SIMS (Flugzeit-Sekundärionen-Massenspektrometrie), IBM (Ionen­strahl­mikroskopie), mPIXE (hochauflösende protoneninduzierte Röntgenemissions-Spektrometrie) und mRBS (hochauflösende Rutherford-Rückstreu-Spektrometrie) untersucht. Am Ende der 24-monatigen Expositionszeit lag die maximale Lungenbelastung bei 5,88 mg Cerdioxid/Lunge und die durchschnittliche Lungenbelastung bei 4,4 ± 1 mg/Lunge. In allen Konzentrationsgruppen zeigte sich ein linearer Anstieg der Lungenlast in Abhängigkeit von der Expositionszeit (R^2^ = 0,96–0,99) (siehe Tabelle 4). 

Nach der Lunge zeigten sich die höchsten ermittelten Cerdioxid-Konzentrationen mit Werten von bis zu 2400 ± 1000 µg Cerdioxid in den Lungen-assoziierten Lymphknoten (LALN) und kennzeichneten somit den primären Exkretionsweg. Extrapulmonal wurde Cerdioxid in folgenden Organen nach 24 Monaten in absteigender Konzentration nachgewiesen: Leber > Niere, Milz >>> Herz > Gehirn > Bulbus olfactorius. Statistisch signifikant erhöhte Werte ergaben sich im Vergleich zu den Kontrollgruppen für die Leber, die Nieren und die Milz (siehe Tabelle 5). Zudem wurde eine lineare Beziehung zwischen den retinierten Dosen in der Lunge (µg/Organ) und der Leber (µg/Organ) mit R^2^ = 0,88 ermittelt.

**Tab.4:** Lungenbelastung und vorhergesagte Clearance-Halbwertszeiten bei Cerdioxid-exponierten Wistar-Ratten (Tentschert et al. 2020)

	Gemessene Lungenbelastung [µg Cerdioxid/Lunge ± SD]			Vorhersage Clearance T_½_ [Tage]^a)^
	3 Monate	12 Monate	24 Monate	
Kontrolle	1,23 ± 0,63	1,56 ± 0,63	1,35 ± 0,36	–
0,1 mg/m^3^	19,92 ± 2,13	49,22 ± 11,46	78,02 ± 34,91	86
0,3 mg/m^3^	48,47 ± 5,65	227,83 ± 27,71	348,44 ± 54,25	114
1,0 mg/m^3^	258,14 ± 4,94	924,36 ± 99,96	1450,07 ± 275,03	164
3,0 mg/m^3^	772,03 ± 126,45	2603,02 ± 337	4411,94 ± 1038,55	200

a) berechnete Werte

SD: Standardabweichung

**Tab.5:** Extrapulmonale Organbelastungen (µg Cerdioxid/Organ) nach 24 Monaten Exposition von Wistar-Ratten (Tentschert et al. 2020)

Organ MW ± SD	Konzentration Cerdioxid [mg/m^3^]			
	0,1	0,3	1	3
Leber	0,30 ± 0,08*	2,63 ±0,39*	3,05 ± 1,06*	11,71 ± 3,38*
Nieren	53,93 ± 7,64*	211,85 ± 75,73*	317,84 ± 86,10*	854,46 ± 386,64*
Milz	19,77 ± 4,18*	75,89 ± 42,59*	281,59 ± 113,19*	793,70 ± 115,20*

MW: Mittelwert; SD: Standardabweichung

*p ≤ 0,05

Relativ hohe Cerdioxid-Werte wurden im Femurknochen sowie im Knochenmark gemessen. Unter der Annahme einer homogenen Verteilung in den Knochen und einem Knochengewicht entsprechend 6 % des Körpergewichts der Ratte wurde die Gesamtbelastung für das Skelett berechnet. Für die Konzentrationsgruppe 3 mg/m^3^ lag damit die Skelettbelastung bei 44,9 µg. Folglich wurden rund 1,1 % der Lungenlast (4412 µg/Lunge) in den Knochen gespeichert. Im Vergleich dazu wurden rund 1,2 % der Lungenlast in allen anderen untersuchten Organen zusammen, ausgenommen die LALN, detektiert. Cerdioxid-Agglomerate und Cerphosphat waren im Knochengewebe eindeutig nachweisbar. Während nanoskalige Partikel in der Nähe des Knochenmarks lagen, befanden sich die größeren Agglomerate in den Knochentrabekeln des spongiösen Knochens. Daher lässt sich annehmen, dass Cerdioxid-Nanopartikel über das Knochenmark in die Knochen gelangen und anschließend als Cerphosphat in Calciumphosphat-haltigen Bereichen mineralisieren (Tentschert et al. 2020). Die von Tentschert et al. (2020) verwendeten Nachweismethoden sind in der Lage, sowohl Cerdioxid als auch Cerphosphat in partikulärer Form nachzuweisen. Die Argumentation von Tentschert et al. (2020) ist logisch formuliert und nachvollziehbar, jedoch bleibt es fraglich, in welcher Form Cerdioxid in die Knochen gelangt und dort als Cerphosphat eingebaut wird. Cerdioxid-Partikel wirken im biologischen System vorrangig aufgrund ihrer katalytischen Fähigkeit an der Partikeloberfläche und nicht aufgrund ihrer Löslichkeit toxisch. Statt der von Tentschert et al. (2020) beschriebenen Makrophagen-Überladung bei einer Konzentration von 3 mg/m^3^ liegt daher eher ein toxischer Einfluss von Cerdioxid auf die Makrophagen vor.

Nach einmaliger oder wiederholter inhalativer Ganzkörperexposition gegen [^144^Ce]-Cerdioxid-Aerosol für fünf bis 50 Minuten war Cer bis zu einem Jahr nach Expositionsende in der Leber und im Skelett von adulten F344/Crl-Ratten in ansteigender Konzentration in Abhängigkeit von der kumulativen Dosis (max. 230 kBq) und dem Analysezeitpunkt (max. 672 Tage) nachweisbar. Der aktivitätsmediane aerodynamische Durchmesser wurde mit 0,9 bis 2,2 µm mit geometrischen Standardabweichungen von 1,4 bis 2,0 angegeben. In 25 Minuten wurden etwa 10 µg Cerdioxid in den Lungen deponiert. Cer konnte weder in den Nieren noch in der Milz nachgewiesen werden. Die Clearance-Rate wurde durch Ganzkörpermessungen des [^144^Ce]-Cerdioxids ermittelt. Innerhalb der ersten sieben Tage wurden 90 % der [^144^Ce]-Cerdioxid-Belastung eliminiert. Die verbliebenen 7,1 ± 4 % der initialen Dosis wiesen eine Eliminationshalbwertszeit von 32 ± 1,5 Tagen auf, weitere 1,2 ± 0,07 % eine Eliminationshalbwertszeit von 170 ± 4,3 Tagen. Die Clearance-Rate nach inhalativer Exposition gegen [^144^Ce]-Cerdioxid entspricht der anderer unlöslicher Aerosole (Lundgren et al. 1992). Vorrangiges Ziel dieser Studie war die Untersuchung der kumulativen Strahlendosis. Angaben zur eingesetzten Cerdioxid-Konzentration fehlen.

Gegen [^144^Ce]-Cerdioxid-Aerosol mit einem aerodynamischen Durchmesser von 0,11 oder 0,06 µm exponierte Hamster wiesen eine Clearance-Rate von 95 % bzw. 60 % innerhalb von vier Tagen auf. Die unterschiedliche Clearance-Rate beruhte wahrscheinlich auf der unterschiedlichen Partikelgröße, wobei kleinere Partikel eine längere Eliminationshalbwertszeit aufwiesen. Allerdings merkten die Autoren an, dass auch ein Leck in der Inhalationskammer bei der ersten Gruppe als Ursache für diesen Effekt in Frage kommen könnte (Kananpilly und Luna 1975). Für Hunde wurde unter Verwendung von [^144^Ce]-Cerchlorid-Aerosol eine individuelle initiale Clearance-Rate von 35–80 % innerhalb von vier Tagen ermittelt (Boecker und Cuddihy 1974). 

Die im Anschluss an die initiale Clearance des oberen Respirationstrakts einsetzende Clearance der Lunge verläuft langsamer. Die berichteten Halbwertszeiten dieser langsamen Clearance-Phase lagen bei Nagetieren zwischen 100 und 190 Tagen (Lundgren et al. 1974; Morgan et al. 1970; Sturbaum et al. 1970; Thomas et al. 1972). Eine schnelle Clearance-Rate von [^144^Ce] wurde auch in C57BL/6J-Mäusen nach einer 20-minütigen Exposition nur über die Nase gegen [^144^Ce]-Cerdioxid-Aerosol (MMAD 1,35–1,42 µm) und einer lebenslangen Beobachtungszeit ermittelt. Angaben zu den verwendeten Konzentrationen sind nicht vorhanden. Innerhalb der ersten sechs Tage wurden 80–90 % der initialen Cer-Belastung aus den Atemwegen eliminiert (Hahn et al. 1980; Lundgren et al. 1974, 1980).

#### 3.1.2 Oral

Die Resorptionsquote von löslichen radioaktiven Cer-Salzen (Nitrate, Chloride) nach oraler Applikation liegt bei adulten Ratten unter 0,1 % der verabreichten Dosis (k. w. A.). Eine etwas höhere intestinale Resorption von löslichen Cer-Salzen wurde bei gesäugten Jungtieren ermittelt, jedoch verblieb in diesen Fällen das Cer in den intestinalen Zellen und war nicht systemisch verfügbar. Es ist davon auszugehen, dass die systemische Verfügbarkeit von unlöslichem Cerdioxid nach oraler Applikation in keinem Fall höher ist als die der untersuchten löslichen Cer-Salze (ECHA 2022).

Nach oraler Applikation von [^141^Ce]-Cernitrat via Schlundsonde an Sprague-Dawley-Ratten im Alter von null, sieben, 14 oder 26 Tagen (jeweils n = 7–9) wurde die Retention von Cer bestimmt. Verwendet wurde ^141^Ce(NO_3_)_3_ in 3N HNO_3_ mit einer spezifischen Aktivität von 0,2 mCi/mg Ce. Jeweils 0,1 µCi ^141^Ce in 0,1 ml Kochsalzlösung (pH 2) wurde den Ratten verabreicht, das heißt, die Tiere erhielten 0,5 µg Ce (k. w. A.). Die Resorptionsquote lag für die im Alter von 26 Tagen exponierten Ratten bei 0,004 %. Bei neugeborenen Ratten fiel der Anteil der gemessenen Radioaktivität an der applizierten Radioaktivität von 98 % am ersten Tag auf 29 % am 16. Tag. Die Radioaktivität wurde hier zu fast 100 % am ersten Tag und zu 93 % am 16. Tag im Magen-Darm-Trakt (insbesondere in den oberen zwei Dritteln der Mikrovilli) nachgewiesen (Inaba und Lengemann 1972). 

Eine Resorption von unter 0,1 % vier Tage nach oraler Gabe (via Schlundsonde) von [^144^Ce]-Cernitrat (keine Angabe der Dosis) wurde für adulte weibliche Sprague-Dawley-Ratten ermittelt (Durbin et al. 1956).

Ähnliche Untersuchungsergebnisse wurden auch für die Verwendung von Cerchlorid beschrieben. Die einmalige orale Gabe (via Schlundsonde) von [^141^Ce]-Cerchlorid an gesäugte, juvenile oder adulte Ratten führte zu höheren Resorptionsquoten bei gesäugten im Vergleich zu juvenilen oder adulten Ratten. Der Anteil der gemessenen Radioaktivität (Ganzkörper-Retention) bei Ratten im Alter von null, sieben oder 14 Tagen lag bei 11; 6,5 bzw. 1,5 % der verabreichten Dosis (3–5 µCi ^144^Ce in 0,025 ml Cer(III)-Chlorid-Lösung in HCl; pH 2,5; k. w. A.). Bei juvenilen (21 Tage) bzw. adulten (100 Tage) Ratten lag die gemessene Radioaktivität 14 Tage nach der Exposition nur noch bei 0,08 bzw. 0,018 % der verabreichten Dosis (Shiraishi und Ichikawa 1972). 

Die einmalige intragastrale Applikation von 1000 oder 1163 mg/kg KG [^144^Ce]-Cerchlorid (in Anwesenheit von Citrat) bei männlichen Swiss-ICR-Mäusen führte zu einer Cer-Akkumulation im Magen, im Duodenum und nur minimal in den anderen Organen der Tiere. Eine Ganzkörpermessung wurde nicht vorgenommen, jedoch befanden sich 97–99 % der Radioaktivität in den zwölf untersuchten Geweben im Magen-Darm-Trakt (Magen und Zwölffingerdarm). Das höchste Niveau der Cer-Akkumulation im Magen-Darm-Trakt wurde vier Stunden nach Expositionsende erreicht. Es fiel danach kontinuierlich innerhalb der siebentägigen Beobachtungszeit ab (Stineman et al. 1978). 

Eine altersabhängige Resorption nach einmaliger Gavage von [^144^Ce]-Cerchlorid oder [^144^Ce]-Cercitrat (Dosis nicht ange­geben) wurde auch für 0–6 bzw. 6–24 Stunden alte C_3_H-Mäuse und Sprague-Dawley-Ratten sowie für 6–24 Stunden alte Yorkshire-Ferkel berichtet (Eisele et al. 1980). 

Yorkshire-Ferkel, die am 1. oder 4. Tag nach der Geburt einmal [^144^Ce]-Cerchlorid (200 µCi trägerfreies ^144^Ce-^144^Pr) per Magensonde erhielten, resorbierten jeweils 2,5–8 % der verabreichten Dosis (k. w. A.). Die Resorption am 1. Tag war dreimal höher als am 4. (Mraz und Eisele 1977).

Die höhere Retention von löslichen Cer-Salzen im Magen-Darm-Trakt von jungen Tieren ist wahrscheinlich auf die höhere Pinozytoseaktivität im Darm von jungen Tieren zurückzuführen. Durch eine Vorbehandlung von gesäugten Mäusen mit Glukokortikoiden (Reduktion der Pinozytoseaktivität) konnte die Resorption stark reduziert werden (Kargačin und Landeka 1990). 

Im Anschluss an eine einmalige orale Gabe von Cerdioxid (maximale Dosis 5000 mg/kg KG) wurde eine weißliche Verfärbung der Faeces, wobei es sich vermutlich um die ausgeschiedene ungelöste Substanz handelte, beobachtet. Aufgrund der Schwerlöslichkeit, geringen Resorption und Verteilung sowie dem Fehlen einer offensichtlichen Metabolisierung wurde postuliert, dass das oral gegebene Cerdioxid sehr wahrscheinlich in unveränderter Form eliminiert wurde (ECHA 2022). 

Durch wiederholte orale Gaben von Cerdioxid in Dosen bis zu 1000 mg/kg KG über 42 Tage an männliche Ratten oder über 54 Tage an weibliche Ratten konnten keine Anzeichen einer Toxizität hervorgerufen werden. Zudem blieb eine Auswirkung auf die Reproduktionsfähigkeit oder die Entwicklung der Nachkommen aus. Die Abwesenheit von toxischen Effekten nach oraler Gabe wurde in dieser Studie so interpretiert, dass mikroskaliges Cerdioxid und/oder seine Abbauprodukte weder resorbiert werden noch toxisch sind (ECHA 2022; Kumari et al. 2014 a).

#### 3.1.3 Dermal

In einer Studie gemäß der OECD-Prüfrichtlinie 428 wurde die dermale Aufnahme von eigens synthetisierten 17 nm großen Cerdioxid-Nanopartikeln (Dispersion in synthetischem Schweiß) durch exzidierte intakte oder abradierte ­humane Haut untersucht. Für die Untersuchungen wurden jeweils drei Kammern der Franz-Diffusionszelle mit 0,6 mg Cerdioxid/cm^2^ befüllt. Eine Permeation von Cerdioxid durch die intakte Haut wurde nach einer 24-stündigen Exposi­tion nicht festgestellt. Die Konzentration von Cer in der Rezeptorflüssigkeit war in den Versuchsansätzen mit Cerdioxid-Nano­partikeln identisch mit der Konzentration aus den Versuchen ohne Nanopartikel-Suspensionen. Demnach war keine Permeation durch die Haut nachweisbar. Bei der Verwendung von abradierter Haut wurden gegenüber den Kontrollproben höhere Mengen an Cer in der Rezeptorflüssigkeit gefunden. Die Permeation in die Hautschichten hinein führte bei der Verwendung von intakter Haut zu einer Flächenbelastung von 3,64 ± 0,15 µg/cm^2^ und bei der Verwendung von abradierter Haut zu einer Flächenbelastung von 7,07 ± 0,78 µg/cm^2^. Höhere Cer-Konzentrationen wurden vor allem in der Epidermis nachgewiesen (Mauro et al. 2019). 

Spezielle Studien zur dermalen Resorption von mikroskaligem Cerdioxid stehen nicht zur Verfügung. Die Aufnahme von [^144^Ce]-Cerchlorid über die intakte Haut von Meerschweinchen lag nach einer dreistündigen Exposition unter 0,001 % und stieg bei abradierter Haut auf 4 % an (Inaba und Suzuki-Yasumoto 1979).

In einer Studie gemäß OECD-Prüfrichtlinie 402 führte eine dermale Exposition von Ratten gegen 2000 mg mikroskaliges Cerdioxid/kg KG über 14 Tage nicht zu klinischen Symptomen bei den Tieren (Monnot 1983). Da es sich bei Cerdioxid um eine schwer lösliche anorganische Substanz handelt, wird auch keine signifikante Hautresorption erwartet. 

#### 3.1.4 Weitere Untersuchungen

Bei Untersuchungen der Brustmilch von laktierenden gesunden Frauen aus München (n = 42) und Madrid (n = 26) wurde Cer mittels ICP-MS in Konzentrationen von 5 ng/l bis zu 65 ng/l (Median 13 ng/l) nachgewiesen. Bei der gleichzeitigen Untersuchung der Cer-Plasmakonzentrationen der Frauen aus München lagen alle bis auf zwei Proben unter der Nachweisgrenze von 10 ng/l. Im Vergleich wurden bei den Frauen aus Madrid Werte von 21,6 bis 70,3 ng/l Cer im Plasma ermittelt (Höllriegl et al. 2010).

In einer Studie wurde bei Ratten (drei Tiere/Gruppe) die Cer-Übertragung vom Muttertier auf den Fetus nach intravenöser Gabe ermittelt. Drei Tage nach Verabreichung von [^144^Ce]-Cerchlorid am 9,5., 12,5. oder 18,5. Gestationstag an das Muttertier stieg der [^144^Ce]-Cer-Gehalt von ca. 0,00005 % der injizierten Aktivität pro Embryo/Fetus am 12,5. Gestationstag auf ca. 0,014 % am 21,5. Gestationstag an. Wurde das Cerchlorid einen Monat vor der Konzeption an das Muttertier verabreicht, lag die Retention von [^144^Ce] am 21,5. Gestationstag mit 0,00009 % der injizierten Aktivität pro Embryo/Fetus deutlich niedriger. Im Vergleich zu [^144^Ce]-Cerchlorid kam es drei Tage nach der Verabreichung von [^144^Ce]-Cercitrat an das Muttertier zu einem deutlich höheren (0,0457 % der injizierten Aktivität pro Embryo/Fetus am 21,5. Gestationstag) Übertritt vom Muttertier in den Fetus (Levack et al. 2002).

Einer Gruppe Meerschweinchen wurde am 50. Gestationstag [^144^Ce]-Cerchlorid (ca. 200 µl; 100 kBq) intravenös verabreicht. Am 57. Gestationstag, kurz vor der Geburt, wurden in den Feten 0,05 % der injizierten Aktivität wiedergefunden (Levack et al. 2002).

### 3.2 Metabolismus

Cerdioxid wird nicht metabolisiert. Eine Reduktion von Cer^4+^ zu Cer^3+^ ist möglich.

## 4 Erfahrungen beim Menschen

### 4.1 Einmalige Exposition

Bei Menschen riefen hohe Dosen von Lanthanoid-Oxiden (2 g/kg KG) Schleimhautschädigungen im Verdauungstrakt (evtl. mechanisch bedingt) hervor (IFA 2023*; *Seiler et al. 1988).

Eine hohe akute Toxizität haben anorganische Cer-Verbindungen bei intravenöser oder intraperitonealer Appli­kation gezeigt. Bei Menschen wurden nach Injektion therapeutischer Dosen von Cer-Salzen (3–12 mg/kg KG als Antikoagulantia) toxische Reaktionen wie Schüttelfrost, Fieber, Kopf- und Muskelschmerzen, abdominale Krämpfe, Hämoglobinämie und Hämoglobinurie beobachtet (IFA 2023).

### 4.2 Wiederholte Exposition

Epidemiologisch gesicherte Angaben über eindeutig durch Lanthanoide, im Speziellem durch Cerdioxid, verursachte berufliche Erkrankungen, liegen bislang nicht vor. In sehr frühen Berichten beschriebene Störungen des Allgemeinbefindens (verändertes Wärmeempfinden, Juckreiz, Kopfschmerzen, Übelkeit) bei Exposition gegen Lanthanoid-Dämpfe (keine näheren Angaben) sind in neueren Studien nicht (mehr) beobachtet worden. Es existieren jedoch deutliche Hinweise darauf, dass die bei inhalativer Einwirkung resultierenden Staubeinlagerungen in der Lunge zu gesundheitlichen Schädigungen führen können (IFA 2023). Stark erhöhte Lanthanoid-Konzentrationen im Lungengewebe und in der Bronchialflüssigkeit, vor allem stark erhöhte Cer-Konzentrationen, wurden hauptsächlich bei Personen nachgewiesen, die langfristig gegen Rauch von Lichtbogenlampen exponiert waren, welche u. a. Lanthanoid-Oxide (vor allem Cer-, Lanthan-, Neodymoxid) emittieren. Die auch röntgenologisch sichtbaren Staubeinlagerungen führten z. T. zu keinen funktionellen Beschwerden oder krankhaften Lungengewebsveränderungen. In einigen Fällen wurden jedoch Staublungenerkrankungen (Pneumokoniosen; auch als Cer-Pneumokoniosen bezeichnet) unterschiedlicher Ausprägung diagnostiziert. Die Befunde umfassten Lungenfunktionsstörungen (obstruktiv/restriktiv), Veränderungen im Röntgenbild (knötchenartige Verschattungen) bis hin zu histologisch nachweisbaren schweren Lungenschädigungen (in Einzelfällen Emphysem und interstitielle Fibrose) (IFA 2023).

Bei der Untersuchung eines 60-jährigen Filmvorführers mit einer zwölfjährigen Exposition gegen Seltene-Erden-Stäube zeigten sich eine interstitielle Lungenerkrankung mit Emphysem und eine massive obstruktive Beeinträchtigung. Histologisch wurde eine diffuse interstitielle Lungenfibrose bestätigt. In der Biopsie wurden hohe Konzentrationen von Cer, Lanthan, Neodym, Samarium, Terbium und Ytterbium nachgewiesen. Ein Zusammenhang zwischen der interstitiellen Lungenfibrose und der berufsbedingten Exposition gegen Seltene-Erden-Staub wurde von den Autoren als sehr wahrscheinlich beschrieben (Porru et al. 2001).

Bei einem weiteren Filmvorführer mit einer 25-jährigen beruflichen Exposition gegen Rauch von Kohle-Lichtbogen­lampen wurden histologisch kleine Granula innerhalb von Makrophagen der tracheobronchialen Lymphknoten und von Kupfferschen Sternzellen der Leber nachgewiesen. Die Granula bestanden aus den Oxiden der Seltene-Erden-Elemente Cer, Lanthan und Neodym, welche Hauptbestandteile der Kohle-Lichtbogenstäbe sind. Die Gewebekonzentration war 250–2000-mal höher als bei nichtexponierten Kontrollen. In dem beschriebenen Fall lagen keine klinischen Symptome einer Pneumokoniose vor (Waring und Watling 1990).

Eine 46-jährige Exposition gegen Rauch von Kohle-Lichtbogenlampen führte bei einem Arbeiter zu einer interstitiellen Pneumokoniose. Die Cer-Konzentrationen in der Lunge und in den Lymphknoten lagen bei 167 bzw. 5 µg/g Feuchtgewicht und waren damit 2400- bzw. 53-mal so hoch wie bei nichtexponierten Kontrollen (Pietra et al. 1984; Sabbioni et al. 1982; Vocaturo et al. 1983). In einer Folgestudie am gleichen Patienten wurde festgestellt, dass die Cer-Konzentration im Lungengewebe 2700–207 000-mal so hoch war wie im Urin, im Blut oder in den Nägeln. Hieraus wird eine sehr geringe Mobilisation und Verteilung aus dem Lungengewebe gefolgert (Pietra et al. 1985). 

In der Literatur sind zahlreiche weitere Fälle von interstitiellen Lungenerkrankungen oder Pneumokoniosen im Zusammenhang mit einer Cer-Akkumulation in der Lunge bei Arbeitern nach langanhaltender Exposition gegen Cer-haltigen Rauch oder Cer-haltige Stäube beschrieben (Dufresne et al. 1994; Heuck und Hoschek 1968; Husain et al. 1980; Kappenberger und Bühlmann 1975; Pairon et al. 1994, 1995; Sulotto et al. 1986; Vogt et al. 1986; Yoon et al. 2005). Die Expositionszeit in den oben genannten Fällen betrug zwischen zehn und 46 Jahren, wobei die Exposition in der Regel über den Rauch von Kohle-Lichtbogenlampen erfolgte. Diese Lampen besitzen einen Kern, der zu 46 % aus „Cerium-Oxid” und zu einem geringeren Anteil aus Cerfluorid-Staub oder den anderen Seltene-Erden-Verbindungen (Lanthan, Neodym, Praseodym und Samarium) besteht (Waring und Watling 1990). Wenn der Kern abbrennt, werden dementsprechend vor allem Oxide und zu einem geringeren Anteil Fluoride des Cers und der anderen Seltene-Erden-Elemente emittiert (US EPA 2009). 

Weitere beschriebene Pneumokoniosen stehen im Zusammenhang mit einer Exposition gegen „Ceriumoxid” während des Herstellungsprozesses oder während dessen Verwendung als Schleifmittel (k. w. A.; McDonald et al. 1995). 

Bei einem männlichen Patienten mit einer 35-jährigen Tätigkeit als Schleifer von optischen Linsen mit Cerdioxid-Exposition wurde eine interstitielle Fibrose diagnostiziert. Elektronenmikroskopisch wurden zahlreiche Cer-haltige partikelförmige Ablagerungen in der Lunge nachgewiesen (McDonald et al. 1995). 

Die Höhe der Cer-Exposition wurde jedoch in keiner dieser Studien ermittelt (US EPA 2009).

Zusammenfassend zeigen die Studien, dass die Akkumulation von Cer-Partikeln in der Lunge und im lymphoretikulären System charakteristisch für eine Cer-Pneumokoniose ist. In den meisten Fällen ist eine diffuse interstitielle oder retikulonoduläre Verschattung im Röntgenbild das erste Anzeichen der Erkrankung. Die Lungenfunktion ist bei den betroffenen Personen nicht bis stark eingeschränkt. Vorkommende Verunreinigungen mit Thorium waren quantitativ zu gering, um entsprechende Symptome auszulösen. Zusätzliche “Siliciumdioxid”-Expositionen (fibrogen) bei Glasarbeitern könnten einen Einfluss auf die beschriebenen Effekte gehabt haben, jedoch fehlt dieser Faktor bei Arbeitern, die gegen Lichtbogenlampen-Staub exponiert waren (US EPA 2009).

In einer Fall-Kontroll-Studie (684 Fälle/724 Kontrollen) wurde der Einfluss von chronischer Cer-Belastung, gemessen als Cer-Konzentration im Fußnagel (Neutronenaktivierungsanalyse), auf das Risiko für einen akuten Herzinfarkt (Zeitpunkt des ersten Myokardinfarktes) untersucht. Die durchschnittlichen Cer-Konzentrationen in den Zehennägeln betrugen 186 µg/kg (95-%-KI (Konfidenzintervall): 177–196 µg/kg) bei den Fällen und 173 µg/kg (95-%-KI: 165–182 µg/kg) bei den Kontrollen. Nach Adjustierung betrug das Verhältnis der Cer-Konzentrationen bei Fällen und Kontrollen 1,085; 95-%-KI 1,025–1,149. Das Odds Ratio (OR) der Erkrankung wurde für die Quintile von Cer (Cut-off-Werte: 111, 142, 171 und 257 µg Cer/kg KG) berechnet. Als Einflussfaktoren wurden Rauchen, Body Mass Index (BMI), Bluthochdruck, Diabetes, positive Familienanamnese für koronare Herzerkrankung, β-Carotin, Lycopin, α-Tocopherol, Selen, Quecksilber und Scandium berücksichtigt. Bei Einbeziehung dieser Einflussfaktoren ergaben sich Hinweise auf eine Assoziation zwischen einem erhöhten Risiko für das Auftreten des ersten Myokardinfarktes und einer erhöhten Cer-Konzentration. Die Odds Ratios in den vierten und fünften Quintilen von Cer betrugen im Vergleich zum niedrigsten 2,09 (95-%-KI 1,05–4,16) bzw. 2,81 (95-%- KI 1,21–6,52), der p-Wert für den Trend 0,011 (Gómez-Aracena et al. 2006).

### 4.3 Wirkung auf Haut und Schleimhäute

Hierzu liegen keine Daten vor.

### 4.4 Allergene Wirkung

#### 4.4.1 Hautsensibilisierende Wirkung

Bei einer 48-jährigen Person, welche beruflich regelmäßig beim Schleifen und Polieren von Linsen mit einem Cerdioxid-haltigen Produkt in Kontakt kam, entfärbten sich über zwei Jahre die Fingernägel sowie die paronychialen Bereiche. Im Epikutantest zeigte sie auch beim wiederholten Testen keine Reaktion auf das Cerdioxid-haltige Produkt, reagierte jedoch auf gleichzeitig getestetes p-Phenylendiamin, Merthiolat und p-tert-Butylphenol einer Standardreihe (Rapaport 1982). 

#### 4.4.2 Atemwegssensibilisierende Wirkung 

Hierzu liegen keine Daten vor.

### 4.5 Reproduktionstoxizität

#### 4.5.1 Fertilität

Der mittlere Gehalt von Cer in der Samenflüssigkeit von polnischen Männern, im Alter von 18 bis 50 Jahren, lag bei 41,9 µg/kg Trockengewicht (Bereich: 4,52–167). In dieser Studie konnte eine positive Korrelation zwischen der Konzentration von Cer, Lanthan, Europium, Gadolinium und der gemessenen Calcium-Konzentration nachgewiesen werden (Marzec-Wróblewska et al. 2015).

In der Studie an polnischen Männern korrelierten die Konzentrationen von Cer, Lanthan und Gadolinium positiv mit einer zunehmenden Motilität und dem Anteil normaler Spermatozoen während sie negativ mit der Spermienkonzentration korrelierten. Es blieb jedoch unklar, auf welchem Weg diese Elemente in die Samenflüssigkeit gelangten. In der Studie konnte ein Anstieg der Konzentration von Cer, Lanthan und Gadolinium mit zunehmender Dauer und Menge des Rauchkonsums festgestellt werden. Die Konzentration von Cer, Lanthan, Europium, Gadolinium und Calcium in der Samenflüssigkeit war zudem signifikant niedriger in der Gruppe von Probanden ohne Alkoholkonsum im Vergleich zu der Gruppe von Probanden mit Alkoholkonsum. Ein Einfluss von Cer, Lanthan, Europium und Gadolinium auf die Spermaqualität konnte nicht festgestellt werden (Marzec-Wróblewska et al. 2015). Bemerkenswert war in diesem Zusammenhang die hohe Konzentration von Cer und Lanthan in Innenräumen mit mäßiger bis hoher Tabakrauch-Konzentration (Böhlandt et al. 2012). Tabakrauch als die vorrangige Ursache für Cer und Lanthan in der Luft von Innenräumen wurde auch in weiteren Studien aufgezeigt (Bolte et al. 2008; Fromme et al. 2009; Slezakova et al. 2009). 

#### 4.5.2 Entwicklungstoxizität 

Die These einer Assoziation zwischen dem Gehalt an Seltene-Erden-Elementen (Ce, La, Pr, Nd) im Haar der Mutter und dem Auftreten von Neuralrohrdefekten bei den Nachkommen konnte in einer Studie aus China nicht bestätigt werden (Huo et al. 2017).

Als Teil einer laufenden Fall-Kontroll-Studie in der Shanxi-Provinz in China wurde eine Risikoabschätzung für eine Assoziation für das Auftreten des fetalen Neuralrohrdefektes (NTD) mit Seltene-Erden-Elementen im mütterlichen Serum während der Schwangerschaft bestimmt. Dazu wurden 200 Mütter von Kindern mit NTD und 400 Mütter mit Kindern ohne externe Fehlbildungen in die Studie einbezogen. Die Kontrollmütter waren zur gleichen Zeit in einem ähnlichen Stadium der Schwangerschaft im selben Krankenhaus. Die Diagnosestellung erfolgte während der Schwangerschaft oder bei der Geburt. Auch ein Fall bei einem elektiven Abort wurde berücksichtigt. Die Blutabnahme wurde bei Diagnosestellung oder bei der Geburt vorgenommen. Die Cer-Konzentrationen im Serum von Müttern der von NTD betroffenen Kinder lagen bei einem Median (25.–75. Perzentil) von 0,116 (0,080–0,233) ng/ml und bei den Kontrollmüttern bei 0,090 (0,063–0,130) ng/ml. Die entsprechenden Mittelwerte ± Standard­abweichung betrugen 0,274 ± 0,522 bzw. 0,161 ± 0,336 ng/ml. Die Cer-Konzentrationen wurden in Tertile eingeteilt: erstes Tertil (Referenz): < 0,078, zweites Tertil: 0,078–0,122 und drittes Tertil: > 0,122 ng/ml. Unter Verwendung des logistischen Regressionsmodells wurden OR ermittelt und für maternales Alter, maternaler BMI, maternale Bildung, Schwangerschaftswoche, Verwendung von Folsäure vor der Konzeption und maternale Grippe oder maternales Fieber adjustiert. Dabei ergab sich für NTD ein erhöhtes OR (95-%-KI) von 1,52 (0,70–3,31) im zweiten Tertil bzw. 4,73 (2,08–10,76) im dritten Tertil im Vergleich zum ersten Tertil. Bei rechnerischer Betrachtung der Co-Exposition gegen die anderen neun Seltene-Erden-Elemente wurde keine Assoziation mehr zwischen der Cer-Konzentration und dem Risiko für NTD gefunden (Wei et al. 2020). Wie die Autoren selbst schreiben, findet die kritische Phase der Neuralrohrbildung in der dritten bis vierten Woche der Embryogenese statt. Die Blutproben hingegen stammen von späteren Zeitpunkten der Schwangerschaft. Die Autoren verweisen jedoch auch darauf, dass über die Schwangerschaft (< 28., 28. bis 37., > 37. Schwangerschaftswoche) hinweg die Cer-Konzentrationen im Serum ähnlich hoch sind (Supplemental Data: Table 16). Der Raucherstatus der Schwangeren wurde nicht abgefragt.

In einer weiteren Studie wurde die Assoziation zwischen einer Exposition gegen Seltene-Erden-Elemente während der Schwangerschaft und dem neonatalen Thyroidea-stimulierenden Hormon (TSH)-Spiegel untersucht. Insgesamt wurden 7367 schwangere Frauen aus Wuhan, China, in die Studie einbezogen. Die Konzentrationen von Cer und Ytterbium wurden im Urin mittels ICP-MS gemessen. Im Mittel betrug die Cer- bzw. Ytterbium-Konzentration im mütterlichen Urin 0,060 µg/g Kreatinin bzw. 0,025 µg/g Kreatinin. Eine Verdopplung der Cer- bzw. Ytterbium-Konzentration im mütterlichen Urin war mit einer Verringerung des neonatalen TSH-Spiegels um 4,07 % (95-%-KI: −5,80 – −2,31 %) oder 5,13 % (95-%-KI: −6,93 – −3,30 %) assoziiert (Liu et al. 2019).

### 4.6 Genotoxizität

Hierzu liegen keine Daten vor. 

### 4.7 Kanzerogenität

Hierzu liegen keine Daten vor.

## 5 Tierexperimentelle Befunde und In-vitro-Untersuchungen

### 5.1 Akute Toxizität

#### 5.1.1 Inhalative Aufnahme 

In einer Studie wurde bei F344/N-Ratten die pulmonale Kanzerogenität von β-Teilchenstrahlung (Dosis von 3,6–37 Gy) aus inhaliertem Cerdioxid untersucht. Als Kontrolle dienten gegen stabiles Cerdioxid exponierte Ratten. Den Kontrolltieren wurde Cerdioxid in einer den anderen Gruppen ähnlichen Massenkonzentration einmalig inhalativ verabreicht. Die verabreichte Konzentration wurde von den Autoren nicht näher spezifiziert. Die Tiere wurden lebenslang (mediane Überlebenszeit der weiblichen Tiere = 744 Tage, der männlichen Tiere = 606 Tage) beobachtet. Bei der anschließenden histologischen Untersuchung wurden nichtneoplastische Veränderungen wie Entzündung (5,1 %), Fibrose (5,6 %), Hyperplasie des Alveolarepithels (4,5 %) und Makrophagenhyperplasie (7,1 %) in der Lunge der Kontrolltiere (n = 1049) nachgewiesen (Lundgren et al. 1996). 

In einer Studie zur Ermittlung der akuten Inhalationstoxizität gemäß OECD-Prüfrichtlinie 403 wurden je fünf weibliche und männliche Wistar-Ratten nur über die Nase gegen Cerdioxid in den Konzentrationen 0 oder 5,05 g/m^3^ vier Stunden lang exponiert. Weitere Angaben zur verwendeten Substanz fehlen. Mortalität und klinische Symptome wurden während der Exposition und täglich bis zu 15 Tage nach Expositionsende erfasst. Das Körpergewicht wurde vor der Exposition, am achten und am 15. Tag ermittelt. Todesfälle traten während des Beobachtungszeitraumes nicht auf. Zwei männliche Tiere zeigten kurz nach der Exposition ungefähr 24 Stunden lang eine angestrengte Atmung und ein gesträubtes Fell. Im Vergleich zu den Kontrolltieren gab es keine statistisch signifikante Abweichung des Körpergewichtes. Bei der Nekropsie waren die Lungen aller exponierten Tiere unvollständig kollabiert und zeigten diffuse weißliche Stellen. Die LC_50_ von Cerdioxid nach vier Stunden lag für männliche und weibliche Ratten über 5,05 g/m^3^ (ECHA 2022).

#### 5.1.2 Orale Aufnahme

In einer Studie zur akuten oralen Toxizität gemäß OECD-Prüfrichtlinie 401 wurde Cerdioxid in einer 10%igen wässrigen Gummiarabikum-Suspension jeweils fünf weiblichen und männlichen Sprague-Dawley-Ratten in den Dosierungen 0 (nur Vehikel) oder 5000 mg/kg KG einmalig oral verabreicht. Weitere Angaben zur verwendeten Substanz fehlen. Direkt nach der Verabreichung sowie nach 60 Minuten, zwei und sechs Stunden und anschließend täglich für 14 Tage wurden die Tiere auf klinische Symptome untersucht. Das Körpergewicht wurde am Tag der Applikation sowie danach am ersten, zweiten, vierten, siebten und 14. Tag ermittelt. Während des Beobachtungszeitraums trat keine Mortalität auf. Am ersten Tag wurde eine weißliche Verfärbung der Faeces beobachtet. Im Vergleich zu den Kontrolltieren kam es zu keiner statistisch signifikanten Abweichung des Körpergewichtes. Auch bei der Nekropsie wurden keine Auffälligkeiten verzeichnet (Monnot 1983).

In einer Studie wurde Cerdioxid einmalig in einer Dosis von 1000 mg/kg KG weiblichen Sprague-Dawley-Ratten (fünf bis zehn Tiere pro Gruppe) oral mittels Magensonde verabreicht. Während der 30-tägigen Beobachtungszeit kam es nur zu gelegentlichen Todesfällen. Daher wurde in dieser Studie eine LD_50_ von über 1000 mg/kg KG angenommen (k. w. A.; Bruce et al. 1963).

In einer Studie von Kumari et al. (2014 b) wurden mikroskalige und nanoskalige Cerdioxid-Partikel gemäß OECD-Prüfrichtlinie 420 in Dosierungen von 0, 100, 500 oder 1000 mg/kg KG zur Untersuchung der akuten oralen Toxizität weiblichen Wistar-Ratten (fünf Tiere/Gruppe) einmalig verabreicht. Die Untersuchungen erfolgten 4, 18, 24, 48 oder 72 Stunden nach der Exposition. Bei der Exposition gegen 1000 mg nano-Cerdioxid/kg KG stiegen die Aktivitäten der alkalischen Phosphatase (ALP) nach 24 und 72 Stunden um 31,77 bzw. 36,18 % und die der Lactatdehydrogenase (LDH) um 86,81 bzw. 74,84 % an. Bei der Exposition gegen 500 mg nano-Cerdioxid/kg KG wurde ein statistisch signifikanter Anstieg der ALP-Aktivität (um 14,10 %) ausschließlich nach 24 Stunden ermittelt. Der Glutathion (GSH)-Gehalt in der Leber, den Nieren und im Gehirn war bei der Exposition gegen nano-Cerdioxid dosisabhängig erniedrigt. Die orale Exposition gegen mikroskalige Cerdioxid-Partikel führte im Vergleich zur Kontrollgruppe nicht zu statistisch signifikanten Veränderungen der ALP- oder LDH-Aktivität und nicht zu statistisch signifikanten Veränderungen des GSH-Gehalts in Leber, Nieren oder Gehirn (Kumari et al. 2014 b). 

#### 5.1.3 Dermale Aufnahme

In einer Studie zur akuten dermalen Toxizität gemäß OECD-Prüfrichtlinie 402 wurde Cerdioxid in einer 10%igen wässrigen Gummiarabikum-Suspension einmalig auf die Haut von jeweils fünf weiblichen und männlichen Sprague-Dawley-Ratten in den Dosierungen 0 (nur Vehikel) oder 2000 mg/kg KG okklusiv 24 Stunden aufgetragen. Direkt nach der Applikation sowie nach 60 Minuten, zwei und sechs Stunden und im Anschluss daran täglich für 14 Tage wurden die Tiere auf Hautreaktionen und klinische Symptome untersucht. Das Körpergewicht wurde am Tag der Applikation sowie am ersten, zweiten, vierten, siebten und 14. Tag danach ermittelt. Mortalität, Hautirritationen oder Abweichungen des Körpergewichtes wurden nicht festgestellt. Bei der Nekropsie am 14. Tag wurden keine Auffälligkeiten verzeichnet (Monnot 1983).

#### 5.1.4 Intraperitoneale und intravenöse Aufnahme

Die einmalige intraperitoneale Verabreichung von 1000 mg Cerdioxid/kg KG an weibliche Sprague-Dawley-Ratten blieb während der 30-tägigen Beobachtungszeit ohne besonderen Befund (k. w. A.; Bruce et al. 1963).

#### 5.1.5 Intratracheale Aufnahme 

Eine intratracheal applizierte Cerdioxid-Suspension (50 mg; k. w. A.) führte bei weißen Ratten nach acht Monaten zu leichten Veränderungen in der Lunge und der Milz. Im Zytoplasma von Alveolarmakrophagen lagen Agglomerate der Staubpartikel vor. Zudem zeigte sich eine moderate Proliferation des Lungengewebes sowie der Bronchien mit teilweise erhöhter Lymphozytenzahl sowie leichter Vermehrung des Bindegewebes und der Kollagenfasern. In der Milz wurde eine Anhäufung von Makrophagen und großen mehrkernigen Zellen beobachtet (US EPA 2009).

Die Kurz-, Mittel- und Langzeit-Effekte von unterschiedlich großen Cerdioxid-Partikeln wurden in der Lunge von männlichen Wistar-Ratten untersucht. Cerdioxid-Partikel mit geometrischen Mittelwerten des Durchmessers von 3,9 (GSD 1,93) µm („Ce-C“-Partikel) und 0,20 (GSD 1,20) µm („Ce-F“-Partikel) wurden hierfür einmalig intratracheal in einer Konzentration von 0 oder 34 mg/kg KG appliziert. Die kurz- und mittelfristigen Effekte wurden nach 3, 7, 14, 30, 90 und 180 Tagen untersucht. Zu diesen Zeitpunkten erfolgten eine biochemische Untersuchung der bronchoalveolären Lavageflüssigkeit (BALF) von je fünf Tieren und eine histologische Untersuchung der Lunge von zwei Tieren. Eine histopathologische Untersuchung der Lunge zur Ermittlung der langfristigen Effekte erfolgte nach sechs, zwölf und 18 Monaten. Das Lungengewicht der „Ce-F“-exponierten Tiere war im Beobachtungszeitraum bis zum 180. Tag statistisch signifikant erhöht. Die BALF der „Ce-F“-Gruppen zeigte ab dem siebten bis zum 180. Tag eine opake milchige Trübung sowie einen Anstieg der Entzündungszellen, insbesondere der Neutrophilen. Zudem lag in den „Ce-F“-Gruppen zu allen Zeitpunkten eine statistisch signifikante Erhöhung der Proteinkonzentration und der LDH-Aktivität vor. Histopathologisch wurden „Ce-F“-Agglomerate, Entzündungen und kleine Granulome in der Zeitspanne bis 180 Tage nachgewiesen. In den „Ce-C“-Gruppen ergab die histopathologische Untersuchung auch 18 Monate nach der Exposition keinen Hinweis auf Langzeiteffekte in der Lunge. Hingegen zeigte sich in den „Ce-F“-Gruppen nach 12 und 18 Monaten eine persistierende Aktivierung und ein vermehrter Untergang der Alveolarmakrophagen sowie eine pulmonale alveoläre Proteinose (Toya et al. 2010).

### 5.2 Subakute, subchronische und chronische Toxizität

#### 5.2.1 Inhalative Aufnahme

In einer Studie gemäß OECD-Prüfrichtlinie 413 wurde die inhalative Toxizität von mikroskaligem Cerdioxid an Sprague-Dawley-Ratten (15 Tiere/Geschlecht) 90 Tage lang getestet. Die Exposition gegen Cerdioxid in Konzentrationen von 0 (Luft); 5; 50,5 oder 507,5 mg/m^3^ erfolgte als Trockenpulver-Aerosol (MMAD = 1,8–2,2 µm, GSD = 1,8–1,9) über die Nase sechs Stunden lang täglich an fünf Tagen in der Woche. Angaben zur primären Partikelgröße fehlen. In der Studie wurden folgende Parameter ermittelt: klinische Symptome, Körpergewicht, Futteraufnahme, ­funktionelle Defizite (Functional Observational Battery, FOB), motorische Aktivität, hämatologische, klinisch-biochemische und Urinparameter, ophthalmologische Befunde, makroskopische pathologische Befunde, Organgewichte sowie histopathologische Befunde in ausgewählten Geweben. Während der Studie waren keine behandlungsbedingten Todesfälle oder klinischen Symptome zu verzeichnen. Auffälligkeiten in Bezug auf die Ophthalmologie, klinische Chemie und Urinanalyse sowie auf das Verhalten der Tiere wurden bei keiner der getesteten Konzentrationen beo­b­achtet. Nach 13 Wochen konnte bei weiblichen Tieren, die gegen 507,5 mg Cerdioxid/m^3^ exponiert worden waren, im Rahmen der FOB eine statistisch signifikante Verringerung (17 %) der Griffstärke an den Vordergliedmaßen festgestellt werden. Expositionsbedingte Veränderungen betrafen zudem die hämatologischen Parameter, das Lungen- und Milzgewicht, die makroskopischen pathologischen Befunde sowie die Histopathologie des Respirationstraktes und des lymphoretikulären Systems. Ein statistisch signifikanter Anstieg der absoluten oder relativen Neutrophilenzahl wurde zwischen der 6. und 13. Woche bei weiblichen Ratten ab 5 mg/m^3^ und bei männlichen Ratten ab 50,5 mg/m^3^ beobachtet (siehe Tabelle 6) (Bio-Research Laboratories Ltd 1994; US EPA 2009).

**Tab.6:** Hämatologische Veränderungen bei männlichen und weiblichen Sprague-Dawley-Ratten nach 13-wöchiger inhalativer Cerdioxid-Exposition (Bio-Research Laboratories Ltd 1994; US EPA 2009)

		Cerdioxid-Konzentration [mg/m^3^]			
		0	5	50,5	507,5
**6. Woche**					
Absolut Neutrophile (MW ± SD)	♂	1509 ± 1697	1440 ± 720	2560 ± 882	3088* ± 1162
	♀	724 ± 494	1230 ± 765	1680** ± 651	1328 ± 562
Absolut Lymphozyten (MW ± SD)	♂	10 057 ± 2455	9891 ± 2602	11 478 ± 1944	10 296 ± 3670
	♀	8791 ± 2355	7537 ± 2068	8083 ± 1737	8916 ± 3678
Absolut Eosinophile (MW ± SD)	♂	122 ± 299	102 ± 136	83 ± 125	110 ± 175
	♀	75 ± 119	67 ± 57	92 ± 88	87 ± 104
Relativ Neutrophile (MW ± SD)	♂	11,3 ± 7,5	12,5 ± 5,9	17,7 ± 5,6	22,8* ± 6,8
	♀	7,8 ± 5,7	13,0 ± 6,3	17* ± 6,4	13,3 ± 4,3
Relativ Lymphozyten (MW ± SD)	♂	85,4 ± 8,3	84,6 ± 6,5	79 ± 5,7	73,7* ± 6,9
	♀	90,8 ± 6,0	84,5 ± 6,7	80,1** ± 7,5	84,9 ± 4,5
**13. Woche **					
Absolut Neutrophile (MW ± SD)	♂	1541 ± 909	1638 ± 622	2844* ± 1038	2698* ± 1101
	♀	325 ± 162	1006*** ± 797	1006*** ± 414	1081*** ± 399
Absolut Lymphozyten (MW ± SD)	♂	6243 ± 1575	6254 ± 1521	8481** ± 3009	5238 ± 1910
	♀	3624 ± 1365	3794 ± 1200	3802 ± 1228	3722 ± 1137
Absolut Eosinophile (MW ± SD)	♂	62 ± 94	85 ± 53	175** ± 173	73 ± 98
	♀	47 ± 55	61 ± 55	36 ± 44	32 ± 42
Relativ Neutrophile (MW ± SD)	♂	18,4 ± 6,4	20,1 ± 6,9	25,4 ± 10,9	33,2* ± 9,5
	♀	8,3 ± 4,2	19,1* ± 10,0	19,1* ± 7,8	21,9* ± 6,3
Relativ Lymphozyten (MW ± SD)	♂	77,5 ± 8,7	76,1 ± 7,9	70,7 ± 11,0	62,7* ± 11,0
	♀	85,7 ± 5,5	75,3* ± 10,7	76,9** ± 8,5	73,9* ± 7,1

MW: Mittelwert; SD: Standardabweichung

^*^ p ≤ 0,01; ^**^ p ≤ 0,05; ^***^ p ≤ 0,001 (Dunnett-Test)

Ein statistisch signifikanter Anstieg des absoluten und relativen Lungengewichtes wurde bei männlichen und weiblichen Tieren ab 50,5 mg/m^3^ festgestellt (siehe Tabelle 7).

**Tab.7:** Relative Lungengewichte von männlichen und weiblichen Sprague-Dawley-Ratten nach 13-wöchiger inhalativer Cerdioxid-Exposition (Bio-Research Laboratories Ltd 1994; US EPA 2009)

		Cerdioxid-Konzentration [mg/m^3^]			
		0	5	50,5	507,5
Mittleres relatives Lungengewicht (%) ± SD	♂	0,334 ± 0,03	0,371 ± 0,03	0,528* ± 0,08	1,024* ± 0,16
	♀	0,477 ± 0,04	0,544 ± 0,06	0,697* ± 0,11	1,358* ± 0,14
Veränderung in %	♂	–	11	58	207
	♀	–	14	46	185

SD: Standardabweichung

* p ≤ 0,01 (Dunnett-Test)

Männliche Ratten zeigten bei 507,5 mg/m^3^ zusätzlich ein statistisch signifikant erhöhtes Milzgewicht (siehe Tabelle 8).

**Tab.8:** Relatives Milzgewicht bei männlichen und weiblichen Sprague-Dawley-Ratten nach 13-wöchiger inhalativer Cerdioxid-Exposition (Bio-Research Laboratories Ltd 1994; US EPA 2009)

		Cerdioxid-Konzentration [mg/m^3^]			
		0	5	50,5	507,5
Mittleres relatives Milzgewicht (%) ± SD	♂	0,162 ± 0,02	0,169 ± 0,03	0,178 ± 0,03	0,188* ± 0,03
	♀	0,216 ± 0,03	0,242 ± 0,03	0,226 ± 0,05	0,222 ± 0,03
% Veränderung	♂	–	4	10	16
	♀	–	12	5	3

SD: Standardabweichung

* p ≤ 0,05 (Dunnett-Test)

Bei der Nekropsie fielen Entfärbungen bzw. blasse Areale oder Herde und nicht kollabiertes Parenchym in den Lungen sowohl der männlichen als auch der weiblichen Ratten auf. Ab 50,5 mg/m^3^ wurden die blassen Areale und Entfärbungen in der Lunge bei Tieren beiderlei Geschlechts beobachtet, bei 5 mg/m^3^ ausschließlich bei weiblichen Tieren.

Innerhalb aller Versuchsgruppen lagen Vergrößerungen und/oder blasse Verfärbungen in den Mandibular-, Bronchial-, Mediastinal- und Pankreaslymphknoten vor. Insbesondere die Lunge filtrierende Bronchial- und Mediastinal­lymphknoten waren bei Tieren beiderlei Geschlechts ab 5 mg/m^3^ vergrößert und entfärbt (siehe Tabelle 9).

**Tab.9:** Ergebnisse der pathologischen Untersuchung der Lymphknoten von männlichen und weiblichen Sprague-Dawley-Ratten nach 13-wöchiger inhalativer Cerdioxid-Exposition (Bio-Research Laboratories Ltd 1994; US EPA 2009)

		Cerdioxid-Konzentration [mg/m^3^]			
		0	5	50,5	507,5
**Mandibular**					
Vergrößerung	♂	4/15	2/15	5/15	4/15
	♀	2/15	4/15	3/15	1/15
Verfärbung	♂	–	–	–	–
	♀	–	–	–	–
**Bronchial**					
Vergrößerung	♂	0/15	4/15	15/15	15/15
	♀	0/15	1/15	14/15	15/15
Verfärbung	♂	0/15	13/15	15/15	15/15
	♀	0/15	15/15	15/15	15/15
**Mediastinal**					
Vergrößerung	♂	0/15	2/15	9/15	9/15
	♀	0/15	1/15	8/15	8/15
Verfärbung	♂	0/15	2/15	9/15	10/15
	♀	1/15	10/15	9/15	10/15
**Pankreas**					
** **Vergrößerung	♂	1/15	0/15	0/15	0/15
	♀	2/15	0/15	1/15	0/15
Verfärbung	♂	–	–	–	–
	♀	0/15	0/15	0/15	1/15

Die Autoren bewerteten nur die Veränderungen der Bronchial- und Mediastinallymphknoten als Effekt der vorangegangenen Cerdioxid-Exposition, da die Befunde in den Mandibularlymphknoten auch in den Kontrollen beobachtet wurden. Eine Anhäufung von mikroskopisch kleinem Material fand sich in der Lunge, den Bronchial-, Mediastinal-, Mandibular- und Pankreaslymphknoten sowie in der Trachea, den Bronchien, dem Larynx, der Nasenhöhle, der Leber und der Milz (siehe Tabelle 10). Die Autoren vermuten, dass es sich bei dem Pigment um die Testsubstanz handelt. Korrelierend mit der Pigmentierung der jeweiligen Gewebe wurde in allen exponierten Gruppen eine Hyperplasie des Alveolarepithels der Lunge, eine Metaplasie im Larynx und eine lymphoide Hyperplasie der Lymphknoten sowie der Lunge mit einer deutlichen Konzentrations-Wirkungs-Beziehung festgestellt (Bio-Research Laboratories Ltd 1994; US EPA 2009).

**Tab.10:** Ergebnisse der histopathologischen Untersuchung von männlichen und weiblichen Sprague-Dawley-Ratten nach 13-wöchiger inhalativer Cerdioxid-Exposition (Bio-Research Laboratories Ltd 1994; US EPA 2009)

		Cerdioxid-Konzentration [mg/m^3^]			
		0	5	50,5	507,5
**Kehlkopf**					
Metaplasie	♂	0/15	3/15	9/15*	13/15*
	♀	0/15	3/15	6/15*	9/15*
Pigment-Akkumulation	♂	0/15	6/15*	9/15*	12/15*
	♀	0/15	0/15	7/15*	9/15*
**Lunge**					
Lymphozytäre Hyperplasie	♂	0/15	0/15	0/15	12/15*
	♀	0/15	0/15	1/15	7/15*
alveoläre epitheliale Hyperplasie	♂	0/15	1/15	11/15*	14/15*
	♀	0/15	0/15	5/15*	15/15*
Pigment-Akkumulation	♂	0/15	15/15*	15/15*	15/15*
	♀	0/15	15/15*	15/15*	15/15*
**Bronchiale Lymphknoten**					
Lymphozytäre Hyperplasie	♂	0/15	11/13*	15/15*	15/15*
	♀	0/15	13/15*	15/15*	15/15*
Pigment-Akkumulation	♂	0/15	13/13*	15/15*	15/15*
	♀	0/15	14/15*	15/15*	15/15*
**Mediastinale Lymphknoten**					
Lymphozytäre Hyperplasie	♂	0/0	2/2	9/10	9/9
	♀	0/1	10/10	9/9	9/10
Pigment-Akkumulation	♂	0/0	2/2	8/10	9/9
	♀	0/1	10/10	9/9	9/10
**Mandibuläre Lymphknoten**					
Lymphozytäre Hyperplasie	♂	0/15	0/3	0/5	2/15
	♀	0/15	0/5	0/3	0/15
Pigment-Akkumulation	♂	0/15	0/3	0/5	6/15*
	♀	0/15	0/5	0/3	6/15*
**Bauchspeicheldrüse, Lymphknoten**					
Lymphozytäre Hyperplasie	♂	–	–	–	–
	♀	0/2	0/0	1/1	0/1
Pigment-Akkumulation	♂	–	–	–	–
	♀	0/2	0/0	1/1	1/1
**Milz**					
Pigment-Akkumulation	♂	0/15	0/15	0/15	6/15*
	♀	0/15	0/0	0/0	3/15

*p ≤ 0,05 (Exakter Test nach Fisher)

In einer 28-Tage-Inhalationsstudie wurde Cerdioxid unterschiedlicher Partikelgröße an männlichen und weiblichen Ratten untersucht. Die Tiere wurden für 40 Minuten, zwei oder sechs Stunden täglich an fünf Tagen in der Woche den Cerdioxid-Aerosolen (NM-211: 10,8; NM-212: 11,9; NM-213: 55 mg/m^3^) nur über die Nase ausgesetzt. Jede der vier Gruppen (Kontrolle, niedrig, mittel, hoch) bestand aus fünf männlichen und fünf weiblichen Tieren (Tabelle 11) (Gosens et al. 2014).

**Tab.11:** Dosisäquivalente basierend auf Expositionszeit und Konzentration (Massenkonzentration (mg/m^3^), Partikelzahl (cm^–3^), Oberflächenkonzentration (m^2^/m^3^)) der verwendeten Cerdioxid-Partikel (NM-211: 5–10 nm, NM-212: 40 nm, NM-213: < 5000 nm) (Gosens et al. 2014)

	Niedrig (40 Minuten Exposition)			Mittel (2 Stunden Exposition)			Hoch (6 Stunden Exposition)		
	[mg/m^3^]	[cm^–3^]	[m^2^/m^3^]	[mg/m^3^]	[cm^–3^]	[m^2^/m^3^]	[mg/m^3^]	[cm^–3^]	[m^2^/m^3^]
**NM-211**	1,2	194 444	0,0767	3,5	583 333	0,224	10,8	1 750 000	0,691
**NM-212**	2,5	111 111	0,0679	6,7	333 333	0,182	19,9	1 000 000	0,540
**NM-213**	5,9^a)^	70 556	0,022	18,4^a)^	211 667	0,069	55^a)^	635 000	0,205

a) Expositionsangabe für männliche Tiere. Weibliche Tiere waren geringfügig abweichenden Massenkonzentrationen ausgesetzt (niedrig: 5,7 mg/m^3^, mittel 18,7 mg/m^3^, hoch: 55 mg/m^3^). Für weitere Berechnungen wurden die Rohdaten verwendet.

Die verwendeten Partikel ähnelten sich in ihrem aerodynamischen Durchmesser (siehe Tabelle 12) und entsprachen den Partikeln aus der Studie von Geraets et al. (2012). Die gegen die mikroskaligen Partikel (NM-213) expo­nierten Tiere wurden im Vergleich der größten Massenkonzentration, der kleinsten Partikelanzahl und der ­geringsten Oberflächenkonzentration ausgesetzt. Die bei der Nekropsie gewonnene BALF wurde für die Bestimmung des Gesamtproteins, der ALP, der LDH, der N-Acetylglucosaminidase (NAG), der γ-Glutamyltransferase (γ-GT), Malon­dialdehyd (MDA) und der Superoxiddismutase (SOD) verwendet. Zusätzlich wurden Blut- und pathologische Untersuchungen durchgeführt.

**Tab.12:** Charakteristik der verwendeten Cerdioxid-Aerosole (Gosens et al. 2014)

Charakteristiken	NM-211	NM-212	NM-213
Nominelle Primärpartikelgröße [nm]	5–10	40	< 5000
MMAD nach APS [µm]	1,02 ± 0,04	1,17 ± 0,34	1,40 ± 0,11
	1,82^a)^	2,07^a)^	1,64^a)^
MMAD nach SMPS [µm]	0,276 ± 0,037	0,366 ± 0,058	0,464 ± 0,058
	1,48^a)^	1,56^a)^	1,32^a)^
BET-Oberfläche [m^2^/g]	63,95 ± 0,30	27,15 ± 0,19	3,73 ± 0,01
Zahlenkonzentration [10^6^ Partikel/cm^3^]	1,79 ± 0,41	1,10 ± 0,70	0,68 ± 0,46
Massenkonzentration [mg/m^3^]	10,79 ± 0,82	19,95 ± 13,21	55,00 ± 6,2

Mittelwerte ± Standardabweichung

APS: Aerodynamischer Partikelgrößenanalysator (misst die aerodynamischen Durchmesser von Partikeln basierend auf ihrem Verhalten in einem Luftstrom); BET: Bestimmung der spezifischen Oberfläche mittels Brunauer-Emmett-Teller-Methode; MMAD: massenmedianer Durchmesser; SMPS: Scanning Mobility Particle Sizer (misst die Größenverteilung von Aerosolpartikeln basierend auf ihrer elektrischen Mobilität)

a) geometrische Standardabweichung der Partikelgrößenverteilung

Anzeichen von Toxizität, Verhaltensauffälligkeiten oder Mortalität traten nicht auf. Die pathologische Untersuchung ergab weiße Verfärbungen der tracheobronchialen Lymphknoten in den Gruppen der hohen Exposition. In der Lunge, den tracheobronchialen Lymphknoten, der Trachea und im Larynx wurden Aggregate mit braunen oder grünen Partikeln oder Makrophagen mit diesen Partikeln beobachtet. Bei allen getesteten Partikeln war bei männlichen und weiblichen Tieren der hohen Dosisgruppen die Zellzahl in der BALF statistisch signifikant erhöht. Zudem stieg die Anzahl der neutrophilen Granulozyten und der Lymphozyten an. Die Aktivitäten von ALP, γ-GT, LDH und NAG sowie das Gesamtprotein nahmen zu, während die Aktivitäten von SOD und MDA unverändert blieben. Alle drei untersuchten Cerdioxid-Partikel verursachten dosisabhängig eine Entzündung der Lunge. Beim Vergleich der Gefährdung durch die drei Cerdioxid-Partikel ist demnach die Reihenfolge abhängig von der Bezugsgröße. Wird die Massenkonzentration zugrunde gelegt, ist das nanoskalige NM-211 das potenteste Material, bei Zugrundelegung der Oberflächenkonzentration das mikroskalige NM-213-Cerdioxid (Gosens et al. 2014).

Die nanoskaligen Partikel aus der Studie von Gosens et al. (2014) wurden in zwei weiteren Inhalationsstudien mit Ganzkörperexposition verwendet. Weibliche Wistar-Ratten wurden eine (NM-211 oder NM-212) oder vier Wochen (NM-212) jeweils sechs Stunden täglich an fünf Tagen in der Woche gegen Cerdioxid-Aerosole von 0; 0,5; 5 oder 25 mg/m^3^ exponiert. Die Vier-Wochen-Studie wurde gemäß OECD-Prüfrichtlinie 412 durchgeführt. Die Charakteristika der verwendeten Cerdioxid-Partikel sind in Tabelle 13 dargestellt. Der Nachbeobachtungszeitraum betrug bei der 5-Tage-Studie 24 Tage und bei der 4-Wochen-Studie 129 Tage. Die Untersuchung der BALF von fünf Tieren pro Gruppe erfolgte in der 5-Tage-Studie drei sowie 24 Tage nach Beendigung der Exposition und in der 4-Wochen-Studie einen Tag sowie 35 Tage nach Beendigung der Exposition. Eine histopathologische Untersuchung des Respirationstraktes an fünf Tieren pro Gruppe wurde in der 5-Tage-Studie am fünften Expositionstag sowie 21 Tage nach Expositionsende und an zehn Tieren pro Gruppe in der 4-Wochen-Studie zwei sowie 34 Tage nach Expositionsende durchgeführt (Keller et al. 2014).

**Tab.13:** Physikalisch-chemische Charakteristika der verwendeten Cerdioxid-Partikel (Keller et al. 2014)

Endpunkte	Cerdioxid NM-212	Cerdioxid NM-211
Primärpartikel-Durchmesser (TEM)	40 nm	4–15 nm D50: 8.2 nm
Agglomerat-Durchmesser (SEM)	3000–150 000 nm	k. A.
Kristallitgröße (XRD)	40,0 nm	12,5 nm
Kristallitphase (XRD)	Cerianit, kubisches CeO_2_	Cerianit, kubisches CeO_2_
Spezifische Oberfläche (Hg, BET)	30 m^2^/g (Hg), 27 m^2^/g (BET)	33 m^2^/g (Hg), 53 m^2^/g (BET)
Oberflächenchemie (XPS)	Ce (III) 14 %, Ce (IV) 86 %	Ce (III) 22 %, Ce (lV) 78 %
**Oberflächenladung**		
Isoelektrischer Punkt (IEP)	> pH 10 (immer kationisch)	= pH 8,3
Zeta-Potential bei pH 7 (aus elektrophoretischer Mobilität)	+ 42 mV	+ 16 mV
**Dispersibilität **(in Wasser)		
D50	432 nm	2839 nm
durchschnittliche Agglomerationszahl (Zentrifugation)	11	346
**Löslichkeit (ICP-MS) **		
in Wasser	0,002 Gew.-%	< 0,001 Gew.-%
in DMEM/FCS	< 0,001 Gew.-%	< 0,001 Gew.-% (rekristallisiert)
in PSF	< 0,001 Gew.-% (rekristallisiert)	< 0,001 Gew.-%
in PBS	< 0,001 Gew.-%	
in FaSSIF	< 0,001 Gew.-%	
in 0,1 N HCI	0,02 Gew.-% (Reifung)	
Verunreinigungen (TGA XPS)	0–7 % organische Verunreinigungen, als Ester- und Alkylgruppen überwiegend an der Partikeloberfläche	1,6 %, darunter geringe Mengen an Alkylen an der Partikeloberfläche

BET: Bestimmung der spezifischen Oberfläche mittels Brunauer-Emmett-Teller-Methode; DMEM: Dulbecco‘s Modified Eagle‘s Medium; FaSSIE: Nüchternzustands-Darmflüssigkeit (Fasted State Intestinal Fluid); FCS: Fötales Kälberserum; Gew.-%: Gewichts-Prozent; Hg: Bestimmung der spezifischen Oberfläche mittels Quecksilber-Porosimetrie; ICP-MS: induktiv gekoppelte Plasma-Massenspektrometrie (Inductively Coupled Plasma Mass Spectrometry); IEP: Isoelektrischer Punkt; k. A.: keine Angaben; PBS: Phosphat-gepufferte Salzlösung; PSF: Phagolysosomale Simulanzflüssigkeit (Phagolysosomal Simulant Fluid); SEM: Rasterelektronenmikroskopie (Scanning Electron Microscopy); TEM: Transmissionselektronenmikroskopie; TGA: Thermogravimetrische Analyse; XPS: Röntgenphotoelektronenspektroskopie (X-ray Photoelectron Spectroscopy); XRD: Röntgenbeugung (X-ray Diffraction)

In der **5-Tage-Studie** von Keller et al. (2014) wurden am Expositionsende 0,011; 0,1 und 0,53 mg Cer/Lunge bei 0,5; 5 bzw. 25 mg NM-212-Cerdioxid/m^3^ gemessen. Nach 21 Tagen betrugen die Cer-Gehalte noch 0,006; 0,088 und 0,4 mg/Lunge. Beim NM-211-Cerdioxid lag eine halb so hohe Lungenbelastung vor. In der **4-Wochen-Studie** wurden am Ende der Exposition 0,04; 0,52 und 2,62 mg Cer/Lunge bei den Konzentrationen von 0,5; 5 bzw. 25 mg NM-212-Cerdioxid/m^3^ gemessen. Aus diesen Daten wurde bei 0,5 mg NM-212-Cerdioxid/m^3^ eine Halbwertszeit von 40 Tagen berechnet. Ab 5 mg/m^3^ kam es zur Überladung der Lunge. Die Konzentration von 25 mg/m^3^ resultierte in einer Halbwertszeit über 200 Tagen. Drei Tage nach dem Ende der fünftägigen Exposition gegen 25 mg/m^3^ NM-212 oder NM-211 kam es zum relativen und absoluten Anstieg von neutrophilen Granulozyten bei gleichzeitiger relativer Abnahme der Leukozyten im Blut. 

Veränderungen in der BALF zeigten sich sowohl in der 5-Tage- als auch in der 4-Wochen-Studie. In der 5-Tage-Studie kam es ab 0,5 mg/m^3^ zu einem statistisch signifikanten Anstieg der Neutrophilenzahl und des cytokine-induced neutrophil chemoattractant-1 (CINC-1). Ab dieser Konzentration führten die NM-211-Partikel zusätzlich zu einem Anstieg des Chemokins MCP-1 (monocyte chemoattractant protein-1) und des Zytokins M-CSF (macrophage colony-stimulating factor). Eine vollständige (0,5 mg/m^3^) bzw. teilweise (5 und 25 mg/m^3^) Regression konnte 24 Tage nach Expositionsende beobachtet werden. Im Vergleich zur 4-Wochen-Studie zeigte sich eine schnellere Regression der BALF-Parameter in der 5-Tage-Studie. Zellmediatoren, insbesondere MCP-1, waren bei 5 und 25 mg/m^3^ nach vier Wochen in der BALF stärker erhöht als nach fünf Tagen. 

Sowohl in der 5-Tage-Studie (25 mg/m^3^) als auch in der 4-Wochen-Studie (5 bzw. 25 mg/m^3^) waren die Lungengewichte erhöht. Sie kehrten 21 Tage (5-Tage-Studie) bzw. 34 Tage (4-Wochen-Studie) nach Expositionsende wieder auf das Kontrollniveau zurück. In der 4-Wochen-Studie wurde zwei Tage nach Ende der Exposition gegen NM-212 konzen­trationsabhängig ein Anstieg alveolärer Makrophagen beobachtet. In den Alveolen war ab 5 mg/m^3^ eosinophiles granuläres Material verteilt. Beide Befunde waren auch 34 Tage nach Ende der Exposition noch vorhanden. Das Vorkommen der alveolären Histiozytose und des eosinophilen granulären Materials korrelierte mit dem erhöhten Lungengewicht von Tieren, die gegen 5 bzw. 25 mg NM-212-Cerdioxid/m^3^ exponiert worden waren. Im Bronchus-assoziierten lymphatischen Gewebe (BALT) traten bei 25 mg/m^3^ Makrophagenaggregate mit Partikeln auf. Bei einem von zehn Tieren (5 mg/m^3^) und bei fünf von zehn Tieren (25 mg/m^3^) wurde eine multifokale granulomatöse Entzündung diagnostiziert. Cer wurde drei und 65 Tage nach Ende der Exposition gegen 25 mg NM-212-Cerdioxid/m^3^ in der Leber nachgewiesen. Aerosolkonzentrationen von 0,5 mg/m^3^ provozierten keine entzündliche Reaktion in der Lunge. Hingegen lösten 5 und 25 mg Cerdioxid/m^3^ Entzündungsreaktionen in der Lunge aus, die sich innerhalb von vier Wochen zu einer multifokalen granulomatösen Entzündung entwickelten. Die Progression der Entzündung hin zu einer granulomatösen Entzündung war abhängig von der Dauer und der Höhe der Partikel-(Oberflächen)-Last in der Lunge. Insgesamt waren die histologischen Befunde der 5-Tage- und 4-Wochen-Studie ähnlich, wobei die Ausmaße der Veränderungen in der 5-Tage-Studie geringer ausfielen. Das NM-211-Cerdioxid führte wahrscheinlich aufgrund seiner größeren Oberfläche im Vergleich zum NM-212-Cerdioxid zu einer höheren biologischen Aktivität (Keller et al. 2014).

Die Auswirkungen von 0; 0,1; 0,3; 1 oder 3 mg/m^3^ der Cerdioxid-Nanopartikel NM-212 auf die Lunge wurden in einer 90-Tage-Studie gemäß OECD-Prüfrichtlinie 413 untersucht. Hierfür wurden weibliche Wistar-Ratten täglich sechs Stunden lang einmal oder an fünf Tagen pro Woche gegen NM-212 über die Nase 28 oder 90 Tage lang expo­niert. Die Untersuchungen erfolgten am ersten Tag, am 28. Tag sowie an den Tagen 90 + 1; 90 + 28 und 90 + 90 (Exposition + Erholung). Das in diesem Versuch verwendete Cerdioxid ist ein sehr gut charakterisiertes nanoskaliges Material des European Commission Joint Research Centre (JRC) Nanomaterial Repository (Ispra, Italien). NM-212 hat eine primäre Partikelgröße von 28,4 nm, eine mittlere BET-Oberfläche von 27,2 m^2^/g, ist in Wasser unlöslich (< 1 µg/l) und hat eine Reinheit von > 99,5 % (Singh et al. 2014). Die Inhalation von Cerdioxid-Nanopartikeln verursachte eine expositionsdauer- und konzentrationsabhängige Zunahme von Cer in der Lunge von exponierten Tieren. Nach dem Expositionsende war eine Partikeleliminierung in allen Gruppen sichtbar. Die Tiere der niedrigen Konzentrationsgruppen (0,1; 0,3 mg/m^3^) sowie die der mittleren und der hohen Konzentrationsgruppe (1; 3 mg/m^3^) zeigten über den Zeitraum einen ähnlichen Kurvenverlauf der Cer-Konzentration in der Lunge normiert auf die Cer-Konzentration in der Luft, wobei die Elimination bei den höheren Konzentrationen (insbesondere bei 3 mg/m^3^) langsamer erfolgte. Eine leicht reduzierte Clearance-Rate wurde bereits bei 1 mg/m^3^ beobachtet. Die Untersuchung der BALF (fünf Tiere pro Gruppe) ergab eine zeit- und konzentrationsabhängige Zunahme von Entzündungszellen, insbesondere der polymorphkernigen Neutrophilen (PMN). Die Anzahl der PMN und Lymphozyten war durch Exposition gegen 1 bzw. 3 mg Cerdioxid/m^3^ zeitabhängig statistisch signifikant erhöht. Zusätzlich wurden leichte, aber statistisch signifikante Zunahmen des Gesamtproteins, der LDH und der β-Glucuronidase innerhalb der hohen Konzentrationsgruppe ermit­telt. Die histopathologische Untersuchung (zehn Tiere pro Gruppe) der Atmungsorgane von Ratten aus der hohen Expositionsgruppe ergab einen Anstieg der Alveolär/Interstitial-Makrophagen bis zum Ende der Expositionsperiode mit einer anschließenden Translokalisation in die lungennahen Lymphknoten ab dem 28. Tag. Begleitet wurden diese Beobachtungen von alveolären und interstitiellen entzündlichen Zellinfiltraten und einer leichten bronchoalveolären Hyperplasie. Zusätzlich waren Anzeichen einer interstitiellen Fibrose nachweisbar. Der Proliferationsmarker Ki67 stieg 28 Tage nach Ende der Exposition gegen 3 mg/m^3^ auf statistisch signifikante Werte an und verharrte auf dem erhöhten Niveau bis zum Ende der Nachbeobachtungszeit. In dieser Studie gelangten die Cerdioxid-Nanopartikel (NM-212) nach Inhalation bis in den Alveolarbereich der Lunge und induzierten dort eine persistierende Entzündung (NOAEC < 1,0 mg/m^3^). Für weitere Ergebnisse siehe auch Abschnitt 5.6.3 (Schwotzer et al. 2017).

In einer 2-Jahre-Studie mit einer zusätzlichen sechsmonatigen expositionsfreien Nachbeobachtungszeit wurden weibliche Ratten vom Stamm Han: WIST gegen nanoskaliges Cerdioxid (NM-212) in den Konzentrationen 0; 0,1; 0,3; 1 oder 3 mg/m³ gemäß OECD-Prüfrichtlinie 453 ganzkörperexponiert. Die Studie, mit dem Ziel die chronische Toxizität und eventuelle Kanzerogenität von nanoskaligem Cerdioxid zu ermitteln, wurde in verschiedene Projekte aufgeteilt. In einem Projekt wurden der Respirationstrakt sowie alle weiteren nach der OECD-Prüfrichtlinie 453 geforderten Organe der Tiere nach einer zwölfmonatigen Inhalation histopathologisch untersucht. Zusätzlich wurden bis auf die Lunge alle weiteren gemäß Prüfrichtlinie 453 geforderten Organe der Tiere nach 24-monatiger Inhalation bzw. nach 24-monatiger Inhalation und zusätzlicher sechsmonatiger Erholungsphase untersucht. Die Testgruppengröße der nach zwölfmonatiger Exposition untersuchten Tiere lag bei zehn Tieren je Konzentration. Expositionsbedingte Veränderungen wurden zu diesem Untersuchungszeitpunkt ausschließlich im Respirationstrakt beobachtet. Sie betra­fen reaktive bzw. adaptive Befunde, wie eine Akkumulation von partikelbeladenen Makrophagen sowie die Nasenhöhle, den Larynx, die Lunge, die tracheobronchialen und mediastinalen Lymphknoten. Makroskopisch wurden bei allen gegen 3 mg/m^3^ und, mit geringerer Inzidenz, bei den gegen 1 mg/m^3^ exponierten Tieren eine weiß-beige bis weiß-gelbliche Verfärbung und eine moderate Vergrößerung der tracheobronchialen und mediastinalen Lymphknoten festgestellt. In den Nasenhöhlen der Tiere der hohen Konzentrationsgruppe wurde ein expositionsbedingter Anstieg von intraepithelialen eosinophilen Globuli zusammen mit einer minimalen Infiltration von Entzündungszellen beobachtet. In der Lunge wurden konzentrationsabhängig neben einer Ansammlung von partikelbeladenen Makrophagen in alveolären/interstitiellen Bereichen sowie im Bronchial-assoziierten lymphatischen Gewebe (BALT) auch synzytiale Riesenzellen im BALT und eine geringgradige bronchoalveoläre Hyperplasie befundet. Zudem wurden in der Lunge konzentrationsabhängig eine alveoläre/interstitielle Infiltration mit Entzündungszellen, eine multifokale alveoläre/interstitielle granulomatöse Entzündung und eine geringgradige interstitielle Fibrose diagnostiziert. Ausschließlich in der hohen Konzentrationsgruppe wurde bei vier der zehn Tiere eine alveoläre Lipoproteinose festgestellt (siehe Tabelle 14) (Ernst et al. 2018; Schaudien et al. 2018).

**Tab.14:** Inzidenz der durch die Cerdioxid-Exposition bedingten histopathologischen Veränderungen in der Lunge weiblicher Ratten nach zwölfmonatiger Exposition (Schaudien et al. 2018)

	**Cerdioxid-Konzentration [mg/m^3^]**				
	**0**	**0,1**	**0,3**	**1**	**3**
Anzahl der Tiere (♀)	10	10	10	10	10
Makrophagenakkumulation alveolär/interstitiell	0	10*	10*	10*	10*
Makrophagenakkumulation, BALT	0	10*	10*	10*	10*
Riesenzellen, synzytial, BALT	0	0	3	9*	10*
Hyperplasie bronchoalveolär (bronchiolärer Typ)	0	1	2	10*	10*
Infiltration Entzündungszellen alveolär/interstitiell	1	4	10*	10*	10*
Granulomatöse Entzündung alveolär/interstitiell	0	1	3	10*	10*
Interstitielle Fibrose	0	3	4	10*	10*
Lipoproteinose alveolär	0	0	0	0	4
Cholesteringranulome	0	0	0	1	1

*p ≤ 0,001 (Chi-Quadrat-/Fisher-Test, zweiseitig)

BALT: bronchial-assoziiertes lymphatisches Gewebe

Adverse Effekte, wie eine alveoläre/interstitielle Infiltration mit Entzündungszellen, eine multifokale alveoläre/inter­stitielle granulomatöse Entzündung und eine geringgradige interstitielle Fibrose, wenn auch nicht statistisch signifikant, wurden bereits bei einer Expositionskonzentration von 0,1 mg/m^3^ beobachtet. Eine NOAEC wurde von den Autoren aufgrund der Befunde für diesen Untersuchungszeitpunkt (zwölf Monate) in dieser Studie nicht abge­leitet. Expositionsbedingte Veränderungen betrafen nach 24-monatiger Exposition ohne und mit sechsmonatiger Erho­lungsphase ausschließlich den Respirationstrakt. Eine Akkumulation von partikelbeladenen Makrophagen lag in der Lunge, der Nasenhöhle, dem Larynx und in den tracheobronchialen und mediastinalen Lymphknoten zu den Untersuchungszeitpunkten 24 und 30 Monate vor (Schaudien et al. 2018).

Die Lungen der Tiere nach 24-monatiger Inhalation von Cerdioxid-Nanopartikeln bzw. nach anschließender sechsmonatiger Erholungsphase wurden in einem getrennten Projekt untersucht. Die Testgruppengröße lag für beide Untersuchungszeitpunkte bei je 50 Tieren je Konzentration. Die makroskopische Untersuchung der Lungen am Ende der Exposition und nach sechsmonatiger Erholungszeit ergab konzentrationsabhängig oberflächliche weiße oder graue Bezirke mit einem Durchmesser von 1–6 mm, welche mit der Akkumulation von partikelbeladenen Makrophagen korrelierten. Bronchioloalveoläre Hyperplasien (alveolärer oder gemischter Typ) zeigten sich konzentrationsabhängig in Schwere und Inzidenz. Statistisch signifikante Werte wurden in den 3-mg/m^3^-Gruppen beobachtet. Eine alveoläre Bronchiolisation (bronchioalveoläre Hyperplasie, bronchiolärer Typ), charakterisiert durch eine Ausweitung des respi­ratorischen Epithels von den terminalen Bronchiolen in die angrenzenden Alveolen hinein, ist eine Reaktion auf inhalierte Partikel und führt zu einer Erhöhung der Clearance-Rate. Von einem statistisch signifikanten Anstieg der alveolären Bronchiolisation waren alle exponierten Gruppen am Ende der Exposition und Gruppen ab einer Konzentration von 0,3 mg/m^3^ nach sechsmonatiger Erholungszeit betroffen. Der Schweregrad der Ausprägung stieg konzentrationsabhängig ab der 1-mg/m^3^-Gruppe an. Eine Entzündung mit Bildung kleiner (Mikro-)Granulome wurde ausschließlich in exponierten Gruppen beobachtet. Dieser Befund zeigte sich bereits ab einer Konzentration von 0,1 mg/m^3^ konzentrationsabhängig und statistisch signifikant. Weiterhin wurde bereits ab 0,1 mg/m^3^ konzentrationsabhängig eine statistisch signifikante minimale bis geringgradige interstitielle und eine pleurale Fibrose diagnostiziert. Eine konzentrationsabhängige Ansammlung partikelbeladener Makrophagen (alveolär/interstitiell, BALT) war normalerweise mit einer alveolären und interstitiellen Infiltration mit Entzündungszellen assoziiert. Im Wesentlichen bestanden die Infiltrate aus mononukleären Zellen und nur zu einem geringen Teil aus Granulozyten. Cerdioxid indu­zierte eine spezifische granulomatöse Entzündung in der Lunge, bestehend aus Mikrogranulomen und Riesenzellen (Synzytien). Viele dieser Mikrogranulome stellten sich als Immungranulome mit einem Lymphozytensaum dar. Bemerkenswert war, dass diese zum Teil auch in den Kontrollgruppen beobachtete Infiltration mit Entzündungszellen einer großen individuellen Variabilität in Bezug auf die Intensität der Entzündung unterlag. Ungefähr ein Drittel der Tiere der jeweiligen Gruppen zeigte eine sehr schwache Reaktion, während ein Drittel mit einer sehr ausgeprägten Entzündung reagierte. Die Ursache dieser individuellen Reaktion blieb ungeklärt. Weitere histopathologische Befunde waren eine alveoläre Lipoproteinose sowie Cholesteringranulome (siehe Tabelle 15 und 16). Insgesamt unterlagen der Schweregrad und die Inzidenz der Befunde durch alle Gruppen hindurch einer rattenstammspezifischen interindividuellen Variabilität. In den niedrigsten Konzentrationsgruppen war insbesondere der Schweregrad deutlich variabel. Eine entzündliche Reaktion bestand bereits in der niedrigsten Konzentrationsgruppe bis zum Ende der Untersuchung (Ernst et al. 2018).

**Tab.15:** Inzidenz der durch die Cerdioxid-Exposition bedingten histopathologischen Veränderungen in der Lunge weiblicher Ratten nach 24-monatiger Exposition (Ernst et al. 2018)

	Cerdioxid-Konzentration [mg/m^3^]				
	0	0,1	0,3	1	3
Anzahl der Tiere (♀)	50	50	50	50	50
Makrophagenakkumulation alveolär/interstitiell	44	50^a)^	50^a)^	50^a)^	50^a)^
Bronchioloalveoläre Hyperplasie ohne Atypien	6	8	9	14	37***
Alveoläre Bronchiolisation	23	47***	45***	45***	49***
Granulomatöse Entzündung alveolär/interstitiell	0	11***	21***	44***	44***
Infiltration von Entzündungszellen alveolär/interstitiell	46	49	49	50	50
Interstitielle Fibrose	39	49**	49**	50***	50***
Cholesteringranulome	18	7*	13	20	8*
Lipoproteinose alveolär	4	4	6	28***	50***

a) partikelbeladen

*p < 0,05

**p < 0,01

***p < 0,001

**Tab.16:** Inzidenz der durch die Cerdioxid-Exposition bedingten histopathologischen Veränderungen in der Lunge weiblicher Ratten nach 24-monatiger Exposition und sechsmonatiger Erholungszeit (Ernst et al. 2018)

	Cerdioxid-Konzentration [mg/m^3^]				
	0	0,1	0,3	1	3
Anzahl der Tiere (♀)	41	48	49	49	50
Makrophagenakkumulation alveolär/interstitiell	41	48^a)^	49^a)^	49^a)^	50^a)^
Bronchioloalveoläre Hyperplasie ohne Atypien	4	5	7	9	12*
Alveoläre Bronchiolisation	14	22	40***	44***	43***
Granulomatöse Entzündung alveolär/interstitiell	0	5*	17***	18***	26***
Infiltration Entzündungszellen alveolär/interstitiell	28	43***	49***	49***	50***
Interstitielle Fibrose	24	48***	49***	49***	50***
Cholesteringranulome	7	6	5	14	11
Lipoproteinose alveolär	3	5	7	26***	46***

a) partikelbeladen

*p<0,05

**p < 0,01

***p < 0,001

#### 5.2.2 Orale Aufnahme

In einer Studie gemäß OECD-Prüfrichtlinie 422 wurden die allgemeine Toxizität sowie die Reproduktions- und Entwicklungstoxizität von Cerdioxid nach täglicher oraler Gabe per Schlundsonde an jeweils zehn männliche und weibliche Sprague-Dawley-Ratten untersucht. Beginnend zwei Wochen vor der Verpaarung bis nach der Paarung und bei weiblichen Tieren während der Trächtigkeit bis fünf Tage nach der Geburt der Jungtiere wurden Dosierungen von 0 (0,5 % wässrige Methylcellulose-Lösung), 150, 450 oder 1000 mg/kg KG und Tag getestet (mit Ausnahme an den ersten drei Tagen, an denen es durch einen Messfehler zur leichten Überdosierung mit 168, 509 oder 1150 mg/kg KG kam). Nähere Angaben zum verwendeten Material fehlen. Während der Studie traten keine unerwarteten Todesfälle oder klinischen Symptome auf. Körpergewicht und Futteraufnahme waren nicht verändert. Bei der FOB sowie den hämatologischen und blutchemischen Befunden ergaben sich keine Auffälligkeiten. Bei der Nekropsie der Neugeborenen wurden keine Auswirkungen auf die Überlebensrate, die Entwicklung des Körpergewichtes oder die mikro- bzw. makroskopischen Untersuchungsergebnisse festgestellt. Als NOAEL für die parentale Toxizität wurden 1000 mg/kg KG abgeleitet (CiT Safety & Health Research Laboratories 2010). 

In einer weiteren Studie gemäß OECD-Prüfrichtlinie 422 wurden die allgemeine Toxizität sowie die Reproduktions- und Entwicklungstoxizität von Cerdioxid nach täglicher Verabreichung per Schlundsonde an jeweils zwölf männliche und zwölf weibliche Sprague-Dawley-Ratten pro Dosisgruppe untersucht. Beginnend zwei Wochen vor der Verpaarung bis nach der Paarung und bei weiblichen Tieren während der Trächtigkeit bis fünf Tage nach der Geburt der Jungtiere wurden Dosierungen von 0, 100, 300 oder 1000 mg/kg KG und Tag getestet. Insgesamt verblieben die männlichen Tiere somit für 38 Tage und die weiblichen Tiere für 41 Tage in der Studie. Das verwendete Cerdioxid hatte eine polyedrische Form mit einer durchschnittlichen primären Partikelgröße von 14 nm (TEM). Der hydrodynamische Durchmesser betrug 76 nm (DLS) und wies auf eine Agglomerat-Bildung der Cerdioxid-Nanopartikel im deionisierten Wasser hin. Während der Studie traten keine unerwarteten Todesfälle oder klinischen Symptome auf. Es gab keine expositionsbedingten Veränderungen in Bezug auf das Körpergewicht oder die Futteraufnahme. Bei der FOB sowie den hämatologischen und blutchemischen Parametern fanden sich keine expositionsbedingten Auffälligkeiten. Die Untersuchung von Geweben (Blut, Leber, Lunge und Nieren) ergab weder bei den Elterntieren noch bei den Neugeborenen einen erhöhten Cer-Gehalt (Lee et al. 2020).

In einer Studie von Kumari et al. (2014 a) wurde die toxische Wirkung von Cerdioxid nach täglicher Schlundsondengabe über einen Zeitraum von 28 Tagen an Wistar-Ratten untersucht. Die Exposition von jeweils fünf männlichen und fünf weiblichen Tieren pro Gruppe erfolgte in Dosierungen von 0, 30, 300 oder 600 mg/kg KG. Die eingesetzten nanoskaligen Cerdioxid-Partikel (CeO_2_-NP) wiesen eine Größe von 24,2 ± 1,63 nm (TEM) sowie einen mittleren Durchmesser von 191,2 ± 20,25 nm (DLS) und die eingesetzten mikroskaligen Cerdioxid-Partikel (CeO_2_-MP) eine Größe von 3,14 ± 1,29 µm (TEM) auf. Die Proben wurden 24 Stunden nach der letzten Behandlung entnommen. Der festgestellte Rückgang der Futteraufnahme, des Körpergewichtes und der relativen Organgewichte sowohl bei Tieren der CeO_2_-NP-Gruppe als auch bei Tieren der CeO_2_-MP-Gruppe war nicht statistisch signifikant. Der Rückgang der Futteraufnahme lag zwischen 13,0 % und 20,0 % in der NP-Gruppe und zwischen 1,7 % und 12,4 % in der CeO_2_-MP-Gruppe. Gleichfalls bestand ein Rückgang des Körpergewichtes zwischen 10,9 % und 16,7 % in der NP-Gruppe und zwischen 8 % und 10,2 % in der CeO_2_-MP-Gruppe. Bei gegen CeO_2_-MP (600 mg/kg KG) oral exponierten Ratten waren keine statistisch signifikanten histopathologischen Veränderungen in der Leber, den Nieren, der Milz, dem Herzen oder dem Gehirn nachweisbar. Die Aktivität der ALP stieg in den CeO_2_-NP-300- und 600-mg/kg-KG-Dosisgruppen bei den männlichen Tieren statistisch signifikant um 18,31 bzw. 37,48 % und bei den weiblichen Tieren statistisch signifikant um 19,18 bzw. 38,00 % an. Die Aktivität der LDH stieg in den CeO_2_-NP-300- und 600-mg/kg-KG-Dosisgruppen bei den männlichen Tieren statistisch signifikant um 78,56 bzw. 179,53 % und bei den weiblichen Tieren statistisch signifikant um 79,06 bzw. 180,85 % an. Die GSH-Gehalte in der Leber, der Niere und im Gehirn waren in den CeO_2_-NP-300- und 600-mg/kg-KG-Dosisgruppen bei beiden Geschlechtern dosisabhängig erniedrigt. Bei den CeO_2_-MP-Gruppen ergaben sich keine statistisch signifikanten Veränderungen des GSH-Gehaltes der Leber, der Nieren und des Gehirns sowie der LDH- und ALP-Aktivität. Die Zunahme der Cer-Konzentration in den verschiedenen Geweben lag bei den CeO_2_-NP-Gruppen bei allen getesteten Dosen deutlich höher im Vergleich zu den CeO_2_-MP-Gruppen. Bei den Dosierungen von 300 und 600 mg/kg KG und Tag wurde in den CeO_2_-MP-Gruppen eine statistisch signifikante Anreicherung von Cer in der Leber und der Milz beobachtet, während eine statistisch signifikante Anreicherung in den Nieren und im Blut erst ab einer Dosierung von 600 mg/kg KG und Tag festgestellt wurde. Statistisch signifikante Mengen Cer wurden mit dem Urin in der CeO_2_-MP-Gruppe bei der Dosierung von 600 mg/kg KG und Tag ausgeschieden. Im Vergleich zu den CeO_2_-NP-Gruppen wurde in den CeO_2_-MP-Gruppen mehr Cer mit den Faeces der Tiere eliminiert (Kumari et al. 2014 a). 

#### 5.2.3 Dermale Aufnahme

Hierzu liegen keine Daten vor.

#### 5.2.4 Fazit 

Mikroskalige und nanoskalige Cerdioxid-Partikel besitzen sehr ähnliche aerodynamische Durchmesser, gelangen in die lungennahen Lymphknoten und führen konzentrationsabhängig zu einer Entzündung in der Lunge von Ratten (Gosens et al. 2014).

In einer Langzeit-Inhalationsstudie mit nanoskaligen Cerdioxid-Partikeln (NM-212) wurden eindeutig Cerdioxid-Agglomerate und Cerphosphat im Knochengewebe von Ratten nachgewiesen (Tentschert et al. 2020). 

Cerdioxid-Partikel wirken im biologischen System vorrangig aufgrund ihres Redoxpotentials an der Partikeloberfläche, jedoch kann es sich bei dem toxischen Agens auch um lösliche Anteile des Cerdioxids handeln. Grundsätzlich kann die Löslichkeit in einem biologischen System, ähnlich wie bei Bariumsulfat, höher sein als im Wasser (Molina et al. 2019). Die von Tentschert et al. (2020) beschriebene Makrophagen-Überladung bei e^i^ner Konzentration von 3 mg/m^3^ ist eher auf den toxischen Einfluss der gelösten Cerdioxid-Anteile auf die Makrophagen zurückzuführen. Dieser Effekt auf die Makrophagen könnte auch der bei Schwotzer et al (2017) beschriebenen reduzierten Clearance bei 3 mg/m^3^ und der leicht reduzierten Clearance bei 1 mg/m^3^ zugrunde liegen.

In der Langzeit-Studie mit weiblichen Ratten von Ernst et al. (2018) wird in der histopathologischen Untersuchung eine Infiltration mit Entzündungszellen aus hauptsächlich mononukleären Zellen und wenigen Granulozyten beobachtet. Cerdioxid induziert eine spezifische granulomatöse Entzündung in der Lunge, bestehend aus Mikrogranulomen und Riesenzellen (Synzytien). Viele dieser Mikrogranulome stellen sich als Immungranulome mit einem Lymphozytensaum dar (Ernst et al. 2018). Bei einer hohen Belastung („Overload“) mit einem granulären biobeständigen Staub kommt es zu einem Zerfall von Makrophagen und einer Wieder-Freisetzung von Partikeln. Hierdurch würde in einer (sub)­chronischen Studie eine Reaktion entsprechend der akuten Phase angestoßen, d. h. es wäre ein Anstieg bzw. eine Akkumulation von PMNs zu erwarten (Hartwig 2012).

In einer 90-Tage-Studie an Sprague-Dawley-Ratten (Bio-Research Laboratories Ltd 1994; US EPA 2009) lag die ermittelte NOAEC für mikroskaliges Cerdioxid bei < 5 mg/m^3^. In Studien mit nanoskaligen Cerdioxid-Partikeln zeigte sich eine NOAEC von < 1 mg/m^3^ (Schwotzer et al. 2017). In einer umfangreichen 2-Jahre-Inhalationsstudie mit nanoskaligen Cerdioxid-Partikeln an Ratten rief bereits eine Expositionskonzentration von 0,1 mg/m^3^ eine statistisch signifikante granulomatöse Entzündung sowie eine interstitielle Fibrose der Lunge hervor. Insgesamt unterlag der Schweregrad und die Inzidenz der Befunde durch alle Gruppen hindurch einer interindividuellen Variabilität, insbesondere in den niedrigsten Konzentrationsgruppen war die Variabilität des Schweregrades jedoch besonders deutlich ausgeprägt (Ernst et al. 2018; Schaudien et al. 2018). Die LOAEC beträgt somit 0,1 mg Cerdioxid/m^3^ und eine NOAEC wurde nicht erhalten.

### 5.3 Wirkung auf Haut und Schleimhäute

#### 5.3.1 Haut

In einer Studie an sechs Neuseeländer-Kaninchen wurde jeweils 0,5 g Cerdioxid auf die intakte oder abradierte Haut (ca. 2,5 cm^2^) 24 Stunden lang okklusiv aufgetragen. Die Hautstellen wurden direkt im Anschluss sowie 24 und 72 Stunden später nach der Methode von Draize untersucht (Draize et al. 1944). Anzeichen einer Hautirritation wurden nicht festgestellt. Der primäre Hautreizungsindex betrug null. Cerdioxid wurde daher nicht als hautirritativ bewertet (Lambert et al. 1993). 

Die Untersuchung der hautirritativen Wirkung von 0,5 g Cerdioxid auf einem 6 cm^2^ großen Hautareal von jeweils drei männlichen Neuseeländer-Kaninchen über vier Stunden blieb ohne auffälligen Befund. Die Beobachtungszeit betrug in dieser Studie 72 Stunden (ECHA 2022). 

In der bereits in Abschnitt 5.1.3 beschriebenen Studie zur dermalen Toxizität wurden mit 2000 mg Cerdioxid/kg KG in einer 10%igen wässrigen Gummiarabikum-Suspension keine Zeichen einer Hautirritation bei Ratten festgestellt (Monnot 1983).

Zur Untersuchung der Hautirritation wurde unter Verwendung des EpiDerm™ Skin Irritation Test (EPI-200-SIT), eines rekonstruierten humanen epidermalen Modells, eine Studie mit nanoskaligem und mikroskaligem Cerdioxid durchgeführt. Die primäre Partikelgröße des nanoskaligen Cerdioxids lag bei 9 nm, die spezifische Oberfläche bei 94,7 m^2^/g. Das mikroskalige Cerdioxid hatte eine primäre Partikelgröße von 320 nm und eine spezifische Oberfläche von 2,64 m^2^/g. Jeweils 50 mg der Testsubstanzen wurden für die Dauer von 960, 1200 oder 1440 Minuten auf das Testsystem aufgetragen und danach die Zellviabilität (MTT-Assay) und das mittlere irritative Potential (MIP) ermit­telt. Die Zellviabilität lag beim nanoskaligen Cerdioxid nach 960 Minuten bei 119,3 %, nach 1200 Minuten bei 67,5 % und nach 1440 Minuten bei 64,5 %, beim mikroskaligen Cerdioxid bei 92,87 % nach 960 Minuten, bei 97,36 % nach 1200 Minuten und bei 86,23 % nach 1440 Minuten. Der MIP-Wert lag bei beiden Substanzen unter 0,8 (< 0,01 beim nanoskaligen Cerdioxid und 0,003 beim mikroskaligen Cerdioxid), daher wurde für keines der beiden Cerdioxid-Partikel ein hautirritatives Potential in vivo angenommen (Park et al. 2007).

Ein humanes Hautäquivalentmodel (HSEM) aus Keratinozyten wurde eine Stunde lang gegen nanoskaliges Cerdioxid (15–40 nm) in einer Konzentration von 1 mg/ml exponiert und die Zellviabilität mit dem MTT-Assay gemessen. Die ermittelte Viabilität von über 91 % sprach gegen ein hautirritatives Potential von Cerdioxid (Miyani und Hughes 2017).

#### 5.3.2 Auge

In einer Studie an sechs Neuseeländer-Kaninchen wurde jeweils 0,1 g Cerdioxid in den linken Bindehautsack der Tiere appliziert. Bei drei Tieren wurde das Auge direkt nach der Exposition mit Kochsalzlösung für 30 Sekunden gespült. Die Untersuchung der Augen erfolgte nach 24, 48 und 72 Stunden nach der Methode von Draize. Der Draize-Score der nicht gespülten Augen lag nach 24 Stunden bei 8,3; nach 48 Stunden bei 6,3 und nach 72 Stunden bei 1,3. Der Draize-Score der gespülten Augen lag nach 24 Stunden bei 5,7; nach 48 Stunden bei 1,3 und nach 72 Stunden bei null (Maximum 110). Cerdioxid wurde in dieser Studie als minimal irritierend am Auge bewertet (Lambert et al. 1993).

In einer Studie gemäß OECD-Prüfrichtlinie 405 wurde jeweils 0,1 g Cerdioxid in den Bindehautsack von drei männlichen Neuseeländer-Kaninchen appliziert und die Wirkung auf die Augen sieben Tage lang beobachtet. Nähere Angaben zum verwendeten Cerdioxid liegen nicht vor. Die Untersuchung der Augen erfolgte nach einer, 24, 48 und 72 Stunden. Bei einer Gesamtbetrachtung ergab sich eine Reizung der exponierten Augen nach einer, 24 und 48 Stunden mit Scores von 9,33; 1,33 bzw. 0,67 (Maximum 110). Drei Tage nach der Applikation war keine Reizung der Augen mehr feststellbar. Cerdioxid wurde in dieser Studie als minimal irritierend am Auge bewertet (ECHA 2022).

### 5.4 Allergene Wirkung

Parallel zum unter Section 5.4.1.1 beschriebenen PLNA wurde ein IgE-Assay an fünf Ratten mit 300 mg mikroskaligem Cerdioxid/ml in DMSO durchgeführt. Zu Versuchsbeginn wurden jeweils zweimal 50 µl intradermal in das Abdomen injiziert und am 7. Tag erfolgte eine subkutane Injektion (50 µl) in den rechten Hinterlauf. Am 14., 21., 28. und 35. Tag wurde jeweils 1 ml Blut entnommen und Gesamt-IgE im Serum bestimmt. Die IgE-Konzentrationen der behandelten Gruppe waren sehr variabel und es ist keine Zeitabhängigkeit sichtbar (Tabelle 17). Es geht aus den Daten nicht hervor, ob die Proben gepoolt oder einzeln gemessen und anschließend ein Mittelwert gebildet wurde. Am 35. Tag wurden die Lymphknoten untersucht. Bei der mikroskopischen Untersuchung waren Cerdioxidpartikel sichtbar, jedoch bestand kein Zusammenhang mit den gemessenen IgE-Werten. Insgesamt gab es keine eindeutigen Hinweise und der Test wurde als negativ bewertet (ECHA 2022).

**Tab.17:** IgE-Gehalte im Serum im PLNA mit Cerdioxid an Ratten (ECHA 2022)

	Serumkonzentration IgE (Einheit nicht angegeben)			
Dosis	Tag 14	Tag 21	Tag 28	Tag 35
0 mg/ml (Vehikelkontrolle)	620 ± 320	560 ± 110	600 ± 90	840 ± 520
300 mg Cerdioxid/ml	490 ± 60	1700 ± 1700	1100 ± 590	500 ± 120
Positivkontrolle (Trimellitsäureanhydrid 100 mg/ml)	2300 ± 580**	2500 ± 970*	2000 ± 600**	1420 ± 620

*p < 0,05

**p < 0,01

#### 5.4.1 Hautsensibilisierende Wirkung

##### 5.4.1.1 Tierexperimentelle Untersuchungen

Ein Maximierungstest nach OECD-Prüfrichtlinie 406 mit **mikroskaligem Cerdioxid** lieferte ein negatives Test­ergebnis. Die Induktion an 20 Dunkin-Hartley-Meerschweinchen erfolgte durch intradermale Injektion einer 50%igen (G/G) wässrigen Suspension und anschließender epikutaner Applikation von 0,5 g (100 %) Cerdioxid als Feststoff. Die Provokation erfolgte mit 0,5 g (100 %) Cerdioxid als Feststoff, wobei bei vier bzw. fünf der zehn Kontrolltiere zwischen einer und 24 Stunden ein leichtes Erythem (Grad 1) festgestellt wurde. Bei den behandelten Tieren wurden ebenfalls nach einer, sechs, 24 und 48 Stunden leichte bis mäßige Erytheme bei 10, 13, 8 und 3 von 20 Tieren beobachtet. Bei den Vehikelkontrollen traten keine Reaktionen auf. Die Autoren schließen eine positive Hautsensibilisierungsreaktion durch eine histopathologische Untersuchung aus (ECHA 2022). Nähere Angaben zur histopathologischen Untersuchung sind nicht aufgeführt. In dieser Studie wurde offenbar der Feststoff nicht in einem Vehikel aufgenommen und aufgetragen, wie von der Prüfrichtlinie vorgesehen.

Es liegt ein negativer Poplitealer Lymphknoten Assay (PLNA) an weiblichen Brown-Norway-Ratten vor. Die Durch­führung und Auswertung des PLNA ist ähnlich zum Local Lymph Node Assay, wobei beim PLNA nur eine einmalige Injektion erfolgt. Ausgewertet wird die Massenzunahme der Lymphknoten (Gewichtsindex). Ein Gewichtsindex von ca. 1 wird negativ bewertet. Oftmals wird ein Gewichtsindex von 2 positiv bewertet, es gibt jedoch kein universelles Kriterium für eine positive Reaktion. Zusätzlich erfolgt die Bestimmung der Stimulationsindices. Auch hier gibt es keine definierten Grenzwerte für Positivität. Häufig wird ein Stimulationsindex > 5 als positiv bewertet (Descotes et al. 1990; Ravel und Descotes 2005). 

Im vorliegenden PLNA mit fünf Ratten pro Dosis wurden den Tieren am ersten Versuchstag 50 µl einer **mikroskaligen Cerdioxid**-Suspension in DMSO in den Konzentrationen 1, 10 und 100 mg/ml in den Hinterfuß injiziert. Nach sieben Tagen wurde eine ^3^H-Thymidinlösung injiziert, fünf Stunden später wurden beide poplitealen Lymphknoten entnommen, Gewichte ermittelt und die Stimulationindices ermittelt. Die Ergebnisse sind in Tabelle 18 dargestellt. Beim Hauptkriterium, dem Gewichtsindex, wurde keine Erhöhung festgestellt (ECHA 2022). Es zeigten sich zwar erhöhte Werte, jedoch ohne erkennbare Konzentrations-Wirkungs-Beziehung und mit sehr hohen Standardabweichungen. Die Ergebnisse lassen aufgrund der sehr hohen Standardabweichungen keine Aussage zu.

**Tab.18:** Ergebnisse des Poplitealen Lymphknoten Assays (ECHA 2022)

	**Gewichtsindex der poplitealen Lymphknoten**	Stimulationsindex der ^3^H-Thymidin-Inkorporation
0 (Vehikelkontrolle, DMSO)	1,7 ± 0,6	6 ± 9
1 mg/ml	2 ± 2	12 ± 25
10 mg/ml	2 ± 1	9 ± 10
100 mg/ml	1,7 ± 0,6	15 ± 22
Positivkontrolle (Trimellitsäureanhydrid 100 mg/ml)	3,0 ± 1,6	25 ± 25

Es liegt ein negativer LLNA (mit 5-Brom-2-deoxyuridin nach OECD-Prüfrichtlinie 442B) mit **nanoskaligem Cerdioxid** (10–30 nm, hydrodynamischer Durchmesser in 3 % Serum in Dimethylformamid (DMF): 502,4 ± 35,0 nm) an je vier weiblichen BALB/c-Mäusen pro Dosisgruppe vor. Für 25, 50 und 100 % nanoskaliges Cerdioxid in DMF mit 3 % Serum wurden Stimulationsindices von 1,81; 2,15 und 1,86 erreicht. Mit denselben Konzentrationen in DMF mit 0,1 % PF-68 (Dispersant) lagen die Stimulationsindices bei 1,98; 1,69 und 1,04. Somit wurde Positivität bei zwei verschiedenen Vehikeln nicht erreicht (Lee et al. 2021).

Insgesamt wird die dermale Aufnahme von Cerdioxid-Nanopartikeln der benannten Größe bei intakter Haut als ­gering erachtet (vgl. Abschnitt 3.1; Lee et al. 2021; Mauro et al. 2019), sodass eine hautsensibilisierende Wirkung unwahr­scheinlich erscheint*.*

##### 5.4.1.2 Untersuchungen mit New Approach Methods (NAMs)

Im KeratinoSens nach OECD-Prüfrichtlinie 442D, welcher die Aktivierung von Keratinozyten testet, wurde für **Cerdioxid-Nanopartikel** (10–30 nm, hydrodynamischer Durchmesser in Arbeitslösung: 517 ± 42 nm) ein negatives Ergebnis erhalten (Kim et al. 2021). Es liegen jedoch nicht ausreichend Vergleichsdaten zur Untersuchung von Metall­nanopartikeln im KeratinoSens vor, um negative Ergebnisse bewerten zu können. 

Bei der Exposition von humanen MoDC (aus Blutmonozyten differenzierte dendritische Zellen) mit **Cerdioxid-Nano­partikeln** (3–5 nm, hydrodynamischer Durchmesser 10 nm) waren keine Veränderungen der Reifungsmarker (z. B. CD80, CD86 und CD83) zu beobachten, dennoch wurde inflammatorisch wirkendes IL-6 und anti-inflammatorisch wirksames IL-10 nachgewiesen. In einem weiteren In-vitro-T-Zell-Aktivierungstest wurde nur eine geringe T-Zell-Proliferation beobachtet, jedoch konnten Th2-Zytokine (IL-4, IL-5 und IL-10) nachgewiesen werden (Schanen et al. 2013). 

Weitere Untersuchungen liegen nicht vor. Auf Basis dieser einzelnen Untersuchungen ist daher keine Bewertung mittels NAMs möglich.

#### 5.4.2 Atemwegssensibilisierende Wirkung

Weitere Untersuchungen mit nanoskaligem Cerdioxid weisen auf eine Adjuvans-Wirkung bei bestehenden (allergischen) Atemwegserkrankungen hin (Dekkers et al. 2019; Meldrum et al. 2018, 2020). Diese Studien liefern jedoch keine Hinweise auf eine eigenständige sensibilisierende Wirkung von Cerdioxid-Nanopartikeln. 

### 5.5 Reproduktionstoxizität

#### 5.5.1 Fertilität

In einer nach OECD-Prüfrichtlinie 422 durchgeführten Toxizitätsstudie mit Screening-Test auf Reproduktions-/Ent­wicklungstoxizität wurden je zehn männliche und zehn weibliche Sprague-Dawley-Ratten pro Dosisgruppe nach täglicher oraler Gabe von 0, 150, 450 oder 1000 mg/kg KG (Cerdioxid in 0,5 % wässriger Methylcellulose-Lösung) untersucht. Die orale Verabreichung per Schlundsonde erfolgte täglich zwei Wochen vor der Verpaarung, während der Verpaarung bis zur Tötung der männlichen Tiere und während der Trächtigkeit bis fünf Tage post partum bei den weiblichen Tieren. Im Vergleich zu den Kontrollen konnten keine Unterschiede bei der Verpaarung, der Trächtigkeit, der Fruchtbarkeit oder der Reproduktion festgestellt werden. Die Körpergewichte und Anzahl der Nachkommen zeigten keine Abweichungen zu der Kontrollgruppe. Als NOAEL wurden 1000 mg/kg KG und Tag für die Fertilität der Elterntiere festgelegt (siehe Abschnitt 5.2.2; CiT Safety & Health Research Laboratories 2010).

Eine weitere Studie nach OECD-Prüfrichtlinie 422 wurde mit täglicher oraler Gabe von nanoskaligem Cerdioxid an jeweils zwölf männliche und weibliche Sprague-Dawley-Ratten pro Dosisgruppe mit 0, 100, 300 oder 1000 mg/kg KG durchgeführt. Die orale Verabreichung per Schlundsonde erfolgte täglich zwei Wochen vor der Verpaarung, während der Verpaarung bis zur Tötung der männlichen Tiere (insgesamt 38 Tage) und während der Trächtigkeit bis fünf Tage post partum bei den weiblichen Tieren (insgesamt 41 Tage). In dieser Studie konnten keine nennenswerten Veränderungen der Reproduktion von Sprague-Dawley-Ratten oder der Entwicklung ihrer Jungtiere bis zu einer Dosis von 1000 mg nano-CeO_2_/kg KG beobachtet werden (siehe Abschnitt 5.2.2; Lee et al. 2020). Der NOAEL für Fertilität liegt bei 1000 mg/kg KG und Tag, der höchsten Dosis.

In einer Studie wurde der Einfluss von Cerdioxid-Nanopartikeln (CNP) auf Fertilitätsparameter bei 24 Monate alten männlichen Ratten untersucht. Die eigens synthetisierten, mit Citrat beschichteten Cerdioxid-Nanopartikel stellen sich im Elektronenmikroskop (TEM) als schwach aggregierte CNP mit nahezu isotoper Form und einer Größe von 2–5 nm dar. Nach einer Woche der Gewöhnung an die Standarddiät wurden drei Gruppen mit jeweils zehn Tieren gebildet. Die Kontrollgruppe erhielt für weitere zehn Tage Standardfutter. Die Gruppen zwei und drei erhiel­ten zusätzlich zum Standardfutter intragastral 10 % Natriumcitrat oder 1 mg Citrat-beschichtete CNP/kg KG. Die Untersuchung der Tiere erfolge 35 Tage nach Beginn des Experiments, da dies der Dauer der Spermatogenese entspricht. Nach der Verabreichung von CNP zeigte sich bei der Untersuchung eine statistisch signifikante Abnahme des Lipidperoxidations-Produkt-Spiegels im Serum sowie ein Anstieg der Katalase- und SOD-Aktivität. Zusätzlich kam es zu einem Anstieg der Spermienzahl (p < 0,001) und zur Verbesserung der quantitativen Spermienparameter wie Motilität, Lebensfähigkeit und Prozentsatz der Spermien. Sowohl die Kontrollgruppe als auch die Natriumcitrat-Gruppe wiesen diese Veränderungen nicht auf. Beide Gruppen zeigten zudem eine altersbedingte Abnahme der Anzahl aktiver Leydig-Zellen sowie fokale Atrophien der Hodenkanälchen. In der CNP-Gruppe konnte hingegen eine Regeneration der Hodenkanälchen, die Erhöhung der Anzahl und Aktivierung der Leydig-Zellen sowie ein 2,5-facher statistisch signifikanter Anstieg des Serum-Testosterons beobachtet werden. Die Fütterung von Citrat-beschichteten CNP führte möglicherweise zur Reduktion des oxidativen Stresses in den Reproduktionsorganen von älteren männlichen Ratten und somit zur Verbesserung der Fertilitätsparameter (Kobyliak et al. 2015).

In zwei Studien an Kaninchen führte „Cerium-Oxid“ zu einer Erhöhung der Fertilität und einer erniedrigten Mortalität sowie einem verstärkten Wachstum der Nachkommen. Hierbei handelt es sich nicht um toxische Effekte. Es wird ein hormetischer Effekt diskutiert (Adu et al. 2015; Akinmuyisitan et al. 2015).

#### 5.5.2 Entwicklungstoxizität

##### 5.5.2.1 In vivo

In einer Studie, ähnlich wie OECD-Prüfrichtlinie 414, wurde die Entwicklungstoxizität von Cerdioxid nach einmaliger pharyngaler Aspiration am 9,5. Trächtigkeitstag von jeweils fünf weiblichen anästhesierten CD1-Mäusen mit Dosen von 0, 5 oder 20 mg/kg KG im Vergleich zu zehn Tieren der Kontrollgruppe im Jahr 2015 untersucht. Bei der Untersuchung am 18,5. Trächtigkeitstag wurde bei 20 mg/kg KG eine leicht höhere Resorptionsrate und eine geringere Anzahl an Embryonen festgestellt. Diese Beobachtungen waren statistisch nicht signifikant. Die Autoren berichten, dass der Grund für die einmalige Gabe war, dass die Anästhesie selbst die Trächtigkeit beeinträchtigt (ECHA 2022). Die Behandlung umfasst nicht die gesamte Organogenese und es ist keine Untersuchung auf Fehlbildungen beinhaltet. Daher ist die Studie nicht geeignet, das entwicklungstoxische Potential von Cerdioxid zu beurteilen.

Die zwei im vorhergehenden Abschnitt beschriebenen, an Sprague-Dawley-Ratten nach OECD-Prüfrichtlinie 422 durchgeführten Studien ergaben keine Effekte auf die Nachkommen (siehe Abschnitt 5.5.1; CiT Safety & Health Research Laboratories 2010; Lee et al. 2020). Der NOAEL für Perinataltoxizität liegt bei 1000 mg/kg KG und Tag, der höchsten Dosis.

Trächtigen NMRI-Mäusen (sechs Tiere/Gruppe) wurde Cerdioxid in den Dosen 0, 10, 25, 80 oder 250 mg/kg KG jeweils am 7. und am 14. Trächtigkeitstag intraperitoneal in destilliertem Wasser verabreicht. Bei dem in dieser Studie verwendeten Cerdioxid handelte es sich um ein käuflich erworbenes Produkt mit einem Partikeldurchmesser von < 5 µm und einer Dichte von 7,13 g/cm^3^. Untersuchungen zur weitergehenden Charakteristik des verwendeten Cerdioxids liegen nicht vor. In der Studie wurden die Hoden von zwei und sechs Tage alten Nachkommen histologisch und biochemisch untersucht. Zusätzlich erfolgte eine biochemische Untersuchung des Serums von 15 Tage alten Tieren. In der höchsten Dosisgruppe (250 mg/kg KG) zeigte sich bei den zwei Tage alten Mäusen eine statistisch signifikante Reduktion der Anzahl der Spermatogonien sowie der Sertoli- und Leydig-Zellen. In der Gruppe der sechs Tage alten Tiere kam es bei der höchsten Dosis zur statistisch signifikanten Reduktion der Anzahl der Spermatogonien und der Leydig-Zellen. Das, als Maß für die Lipid-Peroxidation im Hodengewebe, gemessene MDA war in den 250-mg/kg-Gruppen im Vergleich zur Kontrollgruppe statistisch signifikant erhöht. Ergebnisse dieser Studie zeigten einen schädlichen Einfluss auf die Entwicklung des Hodengewebes bei Mäusen durch Cerdioxid in hohen Konzentrationen (Nemati et al. 2020 b). Die Verabreichung erfolgte intraperitoneal, daher ist ein direkter Effekt der Testsubstanz auf die Frucht möglich. Die Studie ist daher nicht zur Bewertung der fruchtschädigenden Wirkung geeignet. 

In einer weiteren Studie mit gleichem Versuchsaufbau (intraperitoneale Verabreichung von Cerdioxid in den Dosierungen 0, 10, 25, 80 oder 250 mg/kg KG jeweils am 7. und am 14. Trächtigkeitstag) wurde das Nierengewebe sowie der Serummarker MDA und die Eisenreduktionsfähigkeit (FRAP) bei 15 Tage alten Mäusen untersucht. Die mittleren Gesamtvolumina der Nierenkörperchen, der Glomeruli und der Bowmanschen-Kapsel waren, im Vergleich zu der Kontrollgruppe, in der höchsten Dosisgruppe statistisch signifikant erhöht bei gleichzeitiger statistisch signifikanter Reduktion des mittleren Gesamtvolumens des Bowmanschen-Raums (Nemati et al. 2020 a). Eine signifikante Abnahme der Anzahl der Primordialfollikel in den Ovarien wurde am zweiten und am sechsten Tag nach der Geburt sowie eine statistisch signifikante Abnahme der Anzahl der Primärfollikel in den Ovarien am sechsten Tag nach der Geburt bei 80 mg Cerdioxid/kg KG beobachtet. Bei 10 mg/kg KG kam es zunächst zu einem nicht signifikanten Anstieg der Anzahl der Primordialfollikel in den Ovarien am zweiten und am sechsten Tag nach der Geburt. Zeitgleich konnte eine geringe Zunahme des Körpergewichtes beobachtet werden. Statistisch signifikante Unterschiede von MDA und FRAP wurden nicht ermittelt (Nemati et al. 2021). In den beiden Studien wurde die Substanz intraperitoneal verabreicht, weshalb diese Studien, wie oben bereits erwähnt, nicht zur Bewertung der fruchtschädigenden Wirkung geeignet sind.

Die intravenöse Gabe von Cerdioxid-Nanopartikeln (CeO_2_-NP) in Dosierungen von 0 oder 5 mg/kg KG vom 5.–7. Träch­tigkeitstag führte in der Frühträchtigkeit von BALB/c Mäusen (70 Tiere/Gruppe) zu beeinträchtigten Trächtigkeits­verläufen (fetaler Verlust und Wachstumsverzögerungen) und massiven Dysfunktionen der Plazenta. Die eigens für diese Studie synthetisierten Cerdioxid-Nanopartikel hatten eine Größe von 3 bis 5 nm (FE-TEM) und zeigten die für Cer und Sauerstoff charakteristischen Peaks (EDS). Untersuchungen von jeweils zehn Tieren erfolgten am achten, neunten, zehnten sowie am zwölften Trächtigkeitstag. Die Cerdioxid-Konzentration im Dezidualgewebe der Versuchsgruppen erreichte am neunten Trächtigkeitstag ihr Maximum und fiel im Anschluss langsam wieder ab, während sie bei der Kontrollgruppe zu allen Untersuchungszeitpunkten unter der Nachweisgrenze lag. Am zwölften Trächtigkeitstag der CeO_2_-NP-Tiere zeigte sich eine höhere fetale Resorptionsrate ohne weitere pathologische Befunde in anderen Organen (Herz, Leber, Lunge, Nieren). Die Plazenten der Muttertiere waren insgesamt kleiner, leichter und dunkler verfärbt. Eine nähere Untersuchung des Uterus ergab eine fetale Wachstumsrestriktion sowie fetale Blutungen v. a. in der maternalen Dezidualschicht. Veränderungen zeigten sich zudem in weiteren Schichten der Plazenta. Eine statistisch signifikante Reduktion des Serum-β-hCG-Spiegels konnte in der CeO_2_-NP-Gruppe am zwölften Trächtigkeitstag ermittelt werden. Im Weiteren zeigte sich bei den Untersuchungen dieser Studie, dass eine Cerdioxid-Exposition zu einer abnormalen Invasion und Differenzierung der Zytotrophoblasten, einer Schädigung der Dezidualisierung des Endometriums sowie zu einer Störung der Rekrutierung und Differenzierung von NK-Zellen führt. In der Gruppe mit Spontangeburt war sowohl die Anzahl der Nachkommen pro Wurf als auch deren Körpergewicht im Vergleich zur Kontrollgruppe geringer (Zhong et al. 2020).

Trächtige BALB/c-Mäuse (mindestens sieben Tiere/Gruppe) wurden intravenös gegen nanoskaliges Cerdioxid in Dosierungen von 2,5; 4; 5; 7,5 oder 10 mg/kg KG und Tag (durchschnittliche Partikelgröße 3 bis 5 nm) vom fünften bis zum siebten Trächtigkeitstag exponiert. Am 8., 9., 10. und 12. Trächtigkeitstag wurden Plazenten und Feten untersucht, da dies die Schlüsselperiode der plazentalen Entwicklung bei Mäusen darstellt. Die Dosis von 10 mg/kg KG und Tag erwies sich als letal für die Muttertiere. Ab 5 mg/kg KG und Tag wurden Störungen der plazentalen Entwicklung festgestellt. Die Aktivierung der Autophagie war ab 4 mg/kg KG und Tag zu beobachten. Untersuchungen zum mole­kularen Mechanismus wiesen darauf hin, dass nanoskalige Cerdioxid-Partikel zur Aktivierung der Autophagie in plazentalen Trophoblasten führen. Auf zellulärer Ebene führte nanoskaliges Cerdioxid in vitro zur Inhibierung der Migration und Invasion von HTR-8/SVneo-Zellen und zur Aktivierung der Autophagie über den Rapamycin Komplex1 (mTORC1)-Signalweg (Chen et al. 2022).

Die dreimalige intratracheale Applikation von jeweils 0,1 mg nanoskaligen Cerdioxid-Partikeln am 2,5.; 9,5. und 16,5. Trächtigkeitstag von C57BL6/J-Mäusen führte zur Beeinträchtigung der Lungenentwicklung und zu geringeren Gewichten bei den Nachkommen. Die Autoren schätzten ohne nähere Erläuterungen für die Mäuse eine äquivalente inhalierte Konzentration von 2,8 mg/m^3^ für eine Exposition von acht Stunden pro Tag an fünf Tagen ab. Die Größe der verwendeten Cerdioxid-Partikel ist mit 22,4 ± 0,2 nm und die spezifische Oberfläche mit 42 ± 0,5 m^2^/g angegeben. Die Untersuchung der Plazenten mittels ICP-MS zeigte am 17,5. Trächtigkeitstag eine statistisch signifikant erhöhte Cer-Konzentration von 0,004 (0,001–0,007) µg/g im Vergleich zur Kontrollgruppe (nicht detektierbar), in der fetalen Lunge war kein Anstieg der Cer-Konzentration detektierbar. Morphometrische Untersuchungen der Lunge ergaben sowohl am 14,5. Tag sowie am 49,5. Tag nach der Geburt (zehn Tiere/Gruppe) einen statistisch signifikanten Anstieg des MLI („mean linear intercept“) bei gleichzeitiger Verringerung des RAC („radial alveolar count“). Die veränderten Parameter deuten auf Defekte der Alveolenbildung in der Lunge hin. Zudem verringerte sich bei den Nachkommen die alveoläre Oberfläche und die Anzahl der Alveolen in den Lungen. Die Körpergewichte der Muttertiere wiesen keine Veränderungen auf (Paul et al. 2017). 

##### 5.5.2.2 In vitro

In einer Studie mit primären villösen Zytotrophoblasten aus humaner Plazenta wurde die Auswirkung der Exposition mit Cerdioxid-Nanopartikeln über 24 bis 72 Stunden in Konzentrationen von 0,6 (0,1); 2,5 (0,4); 5 (0,8); 10 (1,6); 20 (3,2); 40 (6,3); 80 (12,6); 160 (25,2); 320 (50,4) oder 640 (101) mg/ml (µg/cm^2^) untersucht. Die verwendeten Plazenten wurden während eines Kaiserschnittes zwischen der 37. und 39. Woche der Amenorrhoe von gesunden Nichtraucherinnen entnommen. Die Cerdioxid-Nanopartikel (NM 212) wurden in Zellkulturmedium dispergiert, Ultraschall-behandelt und umgehend, in der finalen Konzentration, den Zellen zugegeben. Die Ergebnisse zeigten, dass sowohl Zytotrophoblasten als auch Synzytiotrophoblasten in der Lage sind, Cerdioxid-Nanopartikel zu internalisieren. Die metabolische Aktivität der Trophoblasten wurde zeit- und konzentrationsabhängig beeinflusst. Eine Verringerung der Zellviabilität trat ab 80 µg/ml nach 24 Stunden und ab 5 µg/ml nach 48 Stunden auf. Cerdioxid-Nanopartikel induzierten die Caspase 3- und -7-Aktivität (statistisch signifikant ab 10 µg/ml) und die LDH-Freisetzung (signifikant ab 320 µg/ml) ohne Hinweis auf oxidativen Stress. Cerdioxid-Nanopartikel verringerten zudem die Fusionskapazität der Zytotrophoblasten zur Bildung von Synzytiotrophoblasten und senkten die Ausschüttung der Schwangerschaftshormone hCG (humanes Choriongonadotropin), hPL (humanes Plazentalaktogen), PlGF (plazentaler Wachstumsfaktor), P4 (Progesteron) und E2 (Estradiol) konzentrationsabhängig (Nedder et al. 2020).

##### 5.5.2.3 Fazit

Eine pränatale Entwicklungstoxizitätsstudie nach gültiger OECD-Prüfrichtlinie 414, die die gesamte Organogenese abdeckt, liegt nicht vor. Bei C57BL6/J-Mäusen treten nach dreimaliger intratrachealer Applikation von 0,1 mg CeO_2_-NP/Tier (gesamt ca. 15 mg/kg KG bei ca. 0,02 kg KG, die Autoren errechnen ohne nähere Angaben eine äquivalente inhalierte Konzentration von 2,8 mg/m^3^ für eine Exposition von acht Stunden pro Tag an fünf Tagen) am 2,5.; 9,5. und 16,5. Trächtigkeitstag bei den Nachkommen Beeinträchtigungen der Alveolenbildung in der Lunge und geringere Gewichte auf (Paul et al. 2017). Da nur eine Dosis getestet wurde, ist die Angabe einer Dosis-Wirkungs-Beziehung nicht möglich. Eine Tabelle mit einer Übersicht zu den Reproduktionsdaten fehlt. Unklar bleiben die Anzahl und das Geschlecht der untersuchten Tiere sowie die Anzahl untersuchter Plazenten bzw. Lungen. Zur Klärung sind weitere Studien statt mit intratrachealer mit inhalativer Gabe und Untersuchung der Lunge notwendig. 

Bei 5 mg CeO_2_-NP/kg KG und Tag kommt es bei dieser Spezies nach intravenöser Gabe vom fünften bis zum siebten oder vom achten bis zum zwölften Trächtigkeitstag zur Beeinträchtigung der plazentalen Entwicklung (Chen et al. 2022; Zhong et al. 2020). In zwei kombinierten, gemäß der OECD-Prüfrichtlinie 422 durchgeführten Toxizitätsstudien inklusive Screening-Test auf Reproduktions-/Entwicklungstoxizität, treten nach wiederholter Verabreichung mittels Schlundsonde an Sprague-Dawley-Ratten bis zur eingesetzten Höchstdosis von 1000 mg Cerdioxid/kg KG und Tag keine Effekte an Muttertieren oder Nachkommen auf (CiT Safety & Health Research Laboratories 2010; Lee et al. 2020).

### 5.6 Genotoxizität

#### 5.6.1 In vitro

In einer Studie an einer humanen Linsenepithelzelllinie (ATCC-LGC CRL-114211) führten Expositionen mit nanoskaligem Cerdioxid in Konzentrationen von 0, 5 oder 10 µg/ml zu keinen statistisch signifikanten Veränderungen in Bezug auf Schwesterchromatidaustausche oder DNA-Schäden im Comet-Assay (Pierscionek et al. 2010).

In einer Kurzzeitstudie wurden (Blut-)Lymphozyten von jeweils vier männlichen und vier weiblichen Spendern (Nichtraucher) gegen nanoskalige Cerdioxid-Partikel in Konzentrationen von 0, 6, 12 oder 18 µg/ml für drei bis 24 Stunden exponiert. Die Größe der verwendeten Cerdioxid-Partikel betrug durchschnittlich 20,7 ± 2,7 nm (SEM, XRD). Die Partikelgröße wurde vom Hersteller mit ≤ 25 nm (BET) angegeben und von den Autoren nicht untersucht. Konzentrationen von 12 oder 18 µg Cerdioxid/ml induzierten in den Lymphozyten nach drei Stunden einen statistisch signifikanten Anstieg an DNA-Schäden im Comet-Assay. Zudem wurde ein statistisch signifikanter Anstieg des γ-H2AX bei allen Konzentrationen ermittelt. Alle drei verwendeten Konzentrationen führten nach 24 Stunden zu einem statistisch signifikanten Anstieg im Mikronukleustest (cytokinesis-block micronucleus assay). Ab der gerings­ten Konzentration wurde eine statistisch signifikante Zytotoxizität mittels Cytokinesis-block proliferation index gemes­sen (Könen-Adıgüzel und Ergene 2018).

In einer Studie an humanen (Blut-)Lymphozyten erwiesen sich sowohl mikro- als auch nanoskalige Cerdioxid-Partikel im Chromosomenaberrationstest und im Mikronukleustest als genotoxisch in allen untersuchten Konzentrationen (0,78–50 µg/l) nach einer Inkubationszeit von 72 Stunden. Die elektronenmikroskopisch bestimmte Größe belief sich auf durchschnittlich 148,25 nm für die mikroskaligen und 25,30 nm für die nanoskaligen Cerdioxid-Partikel. Die verwendeten Lymphozyten wurden von fünf gesunden männlichen Spendern (keine Raucher, kein Alkohol oder Medikamente) gewonnen (Arslan und Akbaba 2020). Die zytotoxische Wirkung der hier verwendeten Cerdioxid-Partikel auf die Lymphozyten wurde nicht bestimmt.

#### 5.6.2 In vivo

In einer 90-Tage-Studie gemäß OECD-Prüfrichtlinie 413 (siehe auch Abschnitt 5.2.1) wurden die Lungen von jeweils sechs weiblichen Wistar-Ratten nach ein-, 28- oder 90-tägiger Exposition über die Nase gegen 0 oder 3 mg Cerdioxid-Nanopartikel (NM-212)/m^3^ immunhistochemisch untersucht. Nach dem Ende der 90-tägigen Exposition konnten statistisch signifikant erhöhte Werte der Genotoxizitäts-Marker γ-H2AX (5 % höher im Vergleich zur Kontrolle) und Hydroxy-2′-desoxyguanosin (8-OHdG) (6 % höher im Vergleich zur Kontrolle) bei allen Untersuchungszeitpunkten (1 Tag, 28 bzw. 90 Tage nach Ende der 90-tägigen Exposition) nachgewiesen werden. Dieser Effekt wurde nach einer 28-tägigen Exposition noch nicht beobachtet (Schwotzer et al. 2017).

Im Rahmen der in den Abschnitten 5.2.1 und 5.7.2 detailliert dargestellten Langzeitstudie gemäß OECD-Prüfrichtlinie 453 zur Untersuchung der chronischen Toxizität und Kanzerogenität von Cerdioxid-Nanopartikeln (NM-212) bei weiblichen Wistar-Ratten wurde auch die Genotoxizität untersucht. Es erfolgte eine histochemische Untersuchung der terminalen Bronchiolen auf die Marker 8-OHdG und γ-H2AX. Die Untersuchung von Lungengewebe (fünf Tiere pro Konzentration) in einer Vorstudie mit einer 28-tägigen Ganzkörperexposition gegen Cerdioxid-Partikel (NM-212) in Konzentrationen von 0; 0,5; 5 oder 25 mg/m^3^ blieb für die gleichen Marker ohne auffälligen Befund. Die Untersuchung von Lungengewebe (zehn Tiere pro Konzentration) in der Hauptstudie mit einer 12-monatigen Ganzkörperexposition gegen Cerdioxid-Partikel (NM-212) in Konzentrationen von 0; 0,1; 0,3; 1 oder 3 mg/m^3^ ergab einen statistisch signifikanten Anstieg des Anteils der γ-H2AX-positiven Zellen ab 0,3 mg/m^3^, jedoch keinen Anstieg des 8-OHdG-Markers (Ernst et al. 2018). Die Phosphorylierung von H2AX geschieht im Zuge der DNA-Reparatur. Meist sind DNA-Doppelstrangbrüche der Auslöser, aber auch Einzelstrangbrüche, z. B. ausgelöst durch UV-Strahlung oder oxidativen Stress (Mishima 2017) und DNA-Schäden während der Proliferation oder der Apoptose können die Phosphorylierung von H2AX bewirken. Die Autoren konnten zudem auch einen möglichen technischen Einfluss (Zeitfaktor) nicht vollständig ausschließen (Ernst et al. 2018). Die erhöhte γ-H2AX-Antikörper-Bindung ist daher nicht ausschließlich ein Nachweis von induzierten Doppelstrangbrüchen, sondern generell von DNA-Schäden. Um zu beweisen, dass es sich zweifelsfrei um DNA-Doppelstrangbrüche handelt, muss die Co-Lokalisation eines zweiten Proteins (z. B. 53BP1) nachgewiesen werden (Rothkamm et al. 2015). Da dies im vorliegenden Fall nicht geschehen ist, wurde hier der Anstieg nicht weiter spezifizierter DNA-Schäden über die γ-H2AX-Quantifizierung nachgewiesen.

In einer Studie wurden mikroskalige- (MP) und nanoskalige- (NP) Cerdioxid-Partikel gemäß OECD-Prüfrichtlinie 420 zur Untersuchung der akuten oralen Toxizität bei weiblichen Wistar-Ratten (fünf Tiere/Gruppe) verwendet. Die Cerdioxid-Partikel wurden in Dosierungen von 0, 100, 500 oder 1000 mg/kg KG einmalig per Schlundsonde verabreicht. Untersuchungen (Comet-Assay, Mikronukleustest und Chromosomenaberrationstest in Blut, Leber und Knochenmark) erfolgten 4, 18, 24, 48 oder 72 Stunden nach Expositionsende. Die mikroskaligen Cerdioxid-Partikel führten unter den beschriebenen Versuchsbedingungen nicht zu signifikanten Veränderungen. Für die nanoskaligen Cerdioxid-Partikel wurden statistisch signifikante Ergebnisse ab einer Dosis von 500 mg/kg KG (Induktion von numerischen Chromosomenaberrationen im Knochenmark) beschrieben. DNA-Schäden (periphere Blutleukozyten, Leber) und Mikronuklei (Knochenmark und peripheres Blut) waren in der Hochdosisgruppe statistisch signifikant erhöht (Kumari et al. 2014 b). Die Studie von Kumari et al. (2014 b) wurde von den Autoren zurückgezogen (Kumari et al. 2022) und wird daher nicht zur Bewertung herangezogen.

In zwei weiteren Inhalationsstudien (siehe auch Abschnitt 5.2.1) wurden jeweils fünf weibliche Wistar-Ratten gegen Cerdioxid-Aerosole in Konzentrationen von 0; 0,5; 5 oder 25 mg/m^3^ eine (NM-211 und NM-212) oder vier Wochen (NM-212) lang jeweils sechs Stunden täglich an fünf Tagen in der Woche ganzkörperexponiert. Die Charakteristika der verwendeten Cerdioxid-Partikel sind in Tabelle 14 dargestellt. Die Zählung der Mikronuklei in den Erythrozyten des peripheren Blutes erfolgte drei und zwei Tage nach Ende der fünftägigen bzw. vierwöchigen Expositionszeit. Alle untersuchten Parameter lagen im Bereich der Kontrollen (Keller et al. 2014).

Die Genotoxizität von mikroskaligen und nanoskaligen Cerdioxid-Partikeln wurde nach 28-tägiger Schlundsondengabe an Wistar-Ratten in Leukozyten des peripheren Bluts sowie in Leber- und Knochenmarkszellen untersucht. Die Exposition von jeweils fünf männlichen und fünf weiblichen Tieren pro Gruppe erfolgte in Dosierungen von 0, 30, 300 oder 600 mg/kg KG und Tag. Die eingesetzten Nanopartikel wiesen eine Größe von 24,2 ± 1,63 nm (TEM) und einen mittleren Durchmesser von 191,2 ± 20,25 nm (DLS) auf, die eingesetzten mikroskaligen Cerdioxid-Partikel eine Größe von 3,14 ± 1,29 µm (TEM). Die Proben wurden 24 Stunden nach der letzten Behandlung gewonnen. Die mikroskaligen Cerdioxid-Partikel führten unter diesen Versuchsbedingungen zu keinen statistisch signifikanten Veränderungen. Expositionen gegen nanoskalige Cerdioxid-Partikel in Dosierungen ab 300 mg/kg KG und Tag führten zu statistisch signifikanten Anstiegen von DNA-Schäden in den Leukozyten und Leberzellen (Comet-Assay), Mikronuklei in Knochenmarkszellen und Leukozyten sowie strukturellen Chromosomenaberrationen in Knochenmarkszellen, wenn die Gaps mitberücksichtigt wurden. Ohne Gaps führte nur die höchste Dosis zu statistisch signifikanten Veränderungen. Das Knochenmark wurde im Mikronukleustest von den Nanopartikeln erreicht, wie die statistisch signifikante Reduzierung der polychromatischen Erythrozyten (PCE) im Verhältnis zu den normochromatischen Erythrozyten (NCE) in der 600-mg/kg-KG-Gruppe zeigte. Der Mitose-Index wurde durch die Exposition gegen Cerdioxid nicht verändert (Kumari et al. 2014 a). Unter der Annahme einer oralen Resorption von 0,1 % und einer inhalativen Aufnahme von 1 % entspricht eine orale Aufnahme beim hier ermittelten NOAEL von 30 mg/kg KG und Tag einer Luftkonzentration von ca. 5,26 mg/m^3^.

Wie bereits erwähnt, wurden im Rahmen der in den Abschnitten 5.2.1 und 5.7.2 detailliert dargestellten Langzeitstudie an weiblichen Wistar-Ratten auch einige Tests zur genotoxischen Wirkung durchgeführt. Nach einer Expositionsdauer von drei oder sechs Monaten wurde Blut von mindestens drei (Expositionszeit 3 Monate) bzw. fünf (Expositionszeit 6 Monate) Tieren pro Konzentrationsgruppe sowohl mikroskopisch als auch durchflusszytometrisch auf die Induktion von Mikronuklei ausgewertet und es erfolgte die durchflusszytometrische Analyse im Pig-a-Assay. Statistisch signifikante genotoxische Befunde wurden mit den verwendeten Tests nicht festgestellt (Cordelli et al. 2017).

#### 5.6.3 Fazit

In-vitro-Daten zur mutagenen Wirkung von mikroskaligem oder nanoskaligem Cerdioxid in Bakterien oder Säuger­zellen liegen nicht vor. Die drei- oder sechsmonatige inhalative Exposition weiblicher Wistar-Ratten hatte bis zur höchsten getesteten Konzentration von 3 mg Cerdioxid-Nanopartikeln/m^3^ im Pig-a-Assay keinen Anstieg von Mutationen in Zellen des peripheren Blutes zur Folge. Mikroskalige Cerdioxid-Partikel wirken nach einmaliger oder wiederholter oraler Gabe nicht klastogen. Nanoskalige Cerdioxid-Partikel führten nach 12-monatiger inhalativer Exposition in der Lunge weiblicher Ratten ab 0,3 mg/m^3^ zu einer Erhöhung der γ-H2AX-Spiegel, jedoch nicht der 8-OHdG-Konzentrationen. Im peripheren Blut wurden nach drei- bzw. sechsmonatiger inhalativer Exposition keine DNA-Schäden (Comet-Assay) bis zur höchsten getesteten Konzentration von 3 mg/m^3^ beobachtet. Mikronukleustests verliefen nach vierwöchiger Exposition gegen bis zu 25 mg/m^3^ und nach drei- und sechsmonatiger inhalativer Expo­sition gegen maximal 3 mg/m^3^ im peripheren Blut negativ. Die 28-tägige orale Gabe führte ab 300 mg/kg KG und Tag im peripheren Blut und in der Leber zu DNA-Schäden (Comet-Assay), zu einem Anstieg Mikronukleus-haltiger Zellen im Knochenmark und Blut sowie zu strukturellen Aberrationen im Knochenmark.

### 5.7 Kanzerogenität

#### 5.7.1 Kurzzeitstudien

Hierzu liegen keine Daten vor.

#### 5.7.2 Langzeitstudien

In einer 24-Monate-Inhalationsstudie mit einer zusätzlichen sechsmonatigen expositionsfreien Nachbeobachtungszeit wurden weibliche Ratten vom Stamm Han: WIST gegen nanoskaliges Cerdioxid (NM-212) in den Konzentrationen 0; 0,1; 0,3; 1 oder 3 mg/m^3^ gemäß OECD-Prüfrichtlinie 453 ganzkörperexponiert (Ernst et al. 2018; Schaudien et al. 2018). Die Studie, in der die chronische Toxizität und Kanzerogenität von nanoskaligem Cerdioxid untersucht werden sollte, wurde in verschiedene Projekte aufgeteilt. Im Rahmen dieser Studie wurde der Respirationstrakt sowie alle weiteren nach OECD-Prüfrichtlinie 453 geforderten Organe der Tiere (zehn Tiere/Konzentration) nach einer zwölfmonatigen Inhalation (Untersuchungszeitpunkt zwölf Monate) histopathologisch untersucht. Zusätzlich wurden bis auf die Lunge alle weiteren gemäß OECD-Prüfrichtlinie 453 geforderten Organe der Tiere nach 24-monatiger Inhalation (Untersuchungszeitpunkt 24 Monate) bzw. nach 24-monatiger Inhalation mit zusätzlicher sechsmonatiger Erholungsphase (Untersuchungszeitpunkt 30 Monate) mit jeweils 50 Tieren pro Konzentrationsgruppe untersucht (Schaudien et al. 2018). Die Lungen der Tiere dieser Studie wurden in einem gesonderten Projekt einer erweiterten histopathologischen Untersuchung unterzogen (Ernst et al. 2018).

Die zwölfmonatige inhalative Exposition gegen Cerdioxid hatte bei den insgesamt 50 Tieren keine expositionsbedingten Neoplasien oder Präneoplasien zur Folge. Zu den Untersuchungszeitpunkten 24 bzw. 30 Monate wurden in den Organen außer den Lungen keine expositionsbedingten Neoplasien oder Präneoplasien beobachtet. Die Anteile der Tiere mit Tumoren in der Kontrollgruppe sowie der gegen 0,1; 0,3; 1 oder 3 mg/m^3^ Cerdioxid exponierten Gruppen lagen bei 87,8 %, 91,0 %, 91,0 %, 89,8 % und 84,0 % (Tabelle 19). Am häufigsten von Tumoren betroffen waren die Hypophyse, die Milchdrüsen, der Uterus und die Leber. Die befundeten Neoplasien entsprachen den üblicherweise in diesem Rattenstamm auftretenden Tumoren und standen in keinem Zusammenhang mit der Exposition (Schaudien et al. 2018).

**Tab.19:** Tumorinzidenzen bei Cerdioxid-exponierten Ratten (Schaudien et al. 2018)

Autor:	Ernst et al. 2018; Schaudien et al. 2018				
Stoff:	nanoskaliges Cerdioxid (NM-212)				
Spezies:	**Ratte**, Han: WIST, 50 ♀ pro Gruppe				
Applikation:	Inhalation				
Konzentration:	0; 0,1; 0,3; 1; 3 mg/m^3^				
Dauer:	24 Monate ohne und mit 6 Monaten Nachbeobachtung				
	**Konzentration [mg/m^3^]**				
	**0**	**0,1**	**0,3**	**1**	**3**
Anzahl untersuchter Tiere (24 u. 30 Mo)	99	98	99	98	100
Anteil der Tiere mit Tumoren (%)	87,8	91,0	91,0	89,8	84,0
Tiere mit gutartigen Tumoren (%)	76,7	81,7	79,7	81,8	77,0
Tiere mit malignen Tumoren (%)	28,2	32,0	34,3	31,3	26,0
Anzahl untersuchter Tiere (24 Mo)	50	50	50	50	50
Bronchioloalveoläres Adenom	0	1	1	1	0
Lungenmetastasen aus Primärtumoren anderer Organe	5	1	5	3	4
Bronchioloalveoläre Hyperplasie mit Atypien	0	3	0	2	5
Bronchioloalveoläre Hyperplasie ohne Atypien	6	8	9	14	37*
	**Konzentration [mg/m^3^]**				
	**0**	**0,1**	**0,3**	**1**	**3**
Anzahl untersuchter Tiere (30 Mo)	49	48	49	49	50
Bronchioloalveoläres Adenom	0	0	1	1	1
Lungenmetastasen aus Primärtumoren anderer Organe	11	9	5	8	4
Bronchioloalveoläre Hyperplasie mit Atypien	1	0	1	0	2
Bronchioloalveoläre Hyperplasie ohne Atypien	4	5	7	9	12*

*p < 0,05

Mo: Monate

Von den Lungen der insgesamt 500 Tiere wurden in einem gesonderten Projekt zu den jeweiligen Untersuchungs­zeitpunkten (24 bzw. 30 Monate) jeweils 60 und mehr Stufenschnitte (3 µm) im Abstand von 500 µm angefertigt und diese histopathologisch untersucht. Zu beiden Untersuchungszeitpunkten gab es keinen statistisch signifikanten Unterschied bei der Anzahl der primären Lungentumore der Expositionsgruppen im Vergleich zur Kontrollgruppe. Nach **24 Monaten **wurden bei jeweils einem Tier der Konzentrationsgruppen 0,1; 0,3 und 1 mg/m^3^ gutartige bronchioloalveoläre Adenome befundet. Lungenmetastasen von Primärtumoren anderer Organe wurden bei 5/50 Tieren der Kontrollgruppe und bei 1/50; 5/50; 3/50 und 4/50 Tieren der Konzentrationsgruppen 0,1; 0,3; 1 und 3 mg/m^3^ nach 24 Monaten beobachtet. Eine erhöhte Inzidenz von Präneoplasien (bronchioloalveoläre Hyperplasien mit Atypien) wurde nicht ermittelt, wohl aber ein konzentrationsabhängiger Anstieg der Hyperplasien ohne Atypien (siehe Tabelle 19). 

Weitere spontane und nicht expositionsbedingte Hyperplasien oder Metaplasien nach 24 Monaten waren fokale Platten­epithel­hyperplasien (1/50 in den 0,1- und 0,3-mg/m^3^-Gruppen), bronchiale/bronchioläre Mukosazellhyperplasien (1/50 bis 2/50 in allen Testgruppen), eine sehr geringgradige pleurale mesotheliale Hyperplasie (1/50 in der 0,3-mg/m^3^-Gruppe), eine Hyperplasie des bronchialrespiratorischen Epithels (1/50 in den Kontroll- und 0,3-mg/m^3^-Gruppen) und eine Metaplasie des bronchialrespiratorischen Plattenepithels (1/50 in der 0,3-mg/m^3^-Gruppe). Zu beachten ist, dass die beiden letzten Veränderungen der 0,3-mg/m^3^-Gruppe ein Tier mit einer durch eine Faseraspiration induzierten Fremd­körper­pneumonie betrafen. Nach **30 Monaten **wurden bei jeweils einem Tier der Konzentrationsgruppen 0,3; 1 und 3 mg/m^3^ gutartige bronchioloalveoläre Adenome befundet. Lungenmetastasen von Primär­tumoren anderer Organe wurden bei 11/49 Tieren der Kontrollgruppe und bei 9/48; 5/49; 8/49 und 4/50 Tieren der Konzen­trationsgruppen 0,1; 0,3; 1 und 3 mg/m^3^ beobachtet. Eine erhöhte Inzidenz an Präneoplasien (bronchioloalveoläre Hyperplasien mit Atypien) wurde nicht ermittelt, wohl aber ein konzentrationsabhängiger Anstieg der Hyperplasien ohne Atypien (siehe Tabelle 19) (Ernst et al. 2018).

**Fazit:** Aus der vorliegenden Langzeitstudie von Ernst et al. (2018) ergibt sich kein Hinweis auf eine kanzerogene Wirkung von Cerdioxid-Nanopartikeln. In Bezug auf Neoplasien wurden nach einer inhalativen Exposition gegen Cerdioxid-Nanopartikel über 24 Monate bzw. 24 Monate plus sechs Monaten Erholungszeit keine statistisch signifikanten Unterschiede zur Kontrollgruppe ermittelt. Die in dieser Studie verwendete Nachweistechnik mit über 60 Schnitten pro Lunge verfügt laut Untersuchungsergebnissen von Kolling et al. (2008) über eine zwei- bis dreifach höhere Detektionsrate im Vergleich zu den standardmäßig verwendeten Untersuchungsmethoden.

### 5.8 Sonstige Wirkungen

Die Zytotoxizität von Cerdioxid wurde in einer In-vitro-Studie mit einer Expositionszeit von 20 Stunden an pulmonalen Alveolarmakrophagen aus Sprague-Dawley-Ratten überprüft. Cerdioxid zeigte sich bei der Untersuchung der Zellviabilität als gering toxisch mit einer LC_50_ (Konzentration, die bei 50 % der Zellen den Zelltod induziert) von 4740 µM. Cerdioxid beeinflusste die Freisetzung von lysosomalen Enzymen (saure Phosphatase, Cathepsin D) nicht. Die morphologische Untersuchung mittels TEM ergab eine Abnahme der Zellen, deren Morphologie im Einklang mit der Kontrollpopulation steht, bei einer laut Autoren geringen Zunahme von Zellen mit einer strukturlosen Oberfläche (Palmer et al. 1987). 

Nano- und mikroskalige Cerdioxid-Partikel (Nano-Ce: 0,009–0,015 µm; Oberfläche 71,3 m^2^/g, Mikro-Ce: 5 µm, Ober­fläche 0,6 m^2^/g) führten in einer Konzentration von 53 µg/cm^2^ oder 100 µl/ml zu einem geringen Anstieg von IL-6 in Beas-2B-Zellen (immortalisierte humane Bronchial-Epithelzellen) und NHBE-Zellen (primäre humane Bronchial-Epithelzellen) (Veranth et al. 2007).

Die Wirkung des Ce^3+^/Ce^4+^-Anteils von Cerdioxid-beschichteten Keramiken auf mesenchymale Knochenstammzellen (BMSC) der Ratte wurde in einer In-vitro-Studie untersucht. Die Ergebnisse der Studie zeigten, dass ein höherer Anteil von Ce^4+^ auch zu größeren Effekten in Bezug auf eine Erhöhung der Zellproliferation, der Aktivität der ALP und der Calciumablagerung führt. Die Beschichtung mit einem höherem Ce^4+^-Anteil förderte die osteogene Differenzierung über den SMAD-abhängigen BMP-Signalweg. Zudem erhöhte sie die Expression von antiinflammatorischen Zytokinen (IL-10, IL-1ra), während sie die Expression von proinflammatorischen Genen (TNF-α, IL-6) sowie die Produktion von reaktiven Sauerstoffspezies in Makrophagen hemmte (You et al. 2017).

## 6 Bewertung

Kritische Effekte sind entzündliche und fibrotische Veränderungen an der Lunge des Menschen und der Ratte.

**MAK-Wert. **Daten beim Menschen zu Cerdioxid deuten auf individuell variierende gesundheitliche Effekte durch das Einatmen von Cer-haltigen Stäuben hin. Fallberichte, in denen Cerdioxid-Partikel noch nach über 15 Jahren in der Lunge nachgewiesen wurden, zeigen eine Cer-Akkumulation in der Lunge und im lymphoretikulären System mit einer sehr hohen Biopersistenz auf (Pairon et al. 1995). Die Zeitspanne seit der letzten Exposition bewegte sich zwischen null und 29 Jahren. Da es sich bei den vorliegenden Humandaten um Fallberichte handelt, können keine Rückschlüsse auf die jeweilige Expositionskonzentration oder mögliche Confounder gezogen werden.

Mit abnehmender Partikelgröße erhöht sich der Anteil der beiden stabilen Cer-Valenzzustände zugunsten des Ce^3+^-Anteils. Dies führt zu veränderten chemisch-physikalischen Eigenschaften, unter anderem zu einem erhöhten Redox­potential (Ce^3+^/Ce^4+^) und einer höheren Löslichkeit (Dekkers et al. 2017). Die Änderungen der chemisch-physikalischen Eigenschaften verlaufen kontinuierlich und erlauben keine eindeutige Diskriminierung zwischen mikro- und nanoskaligen Cerdioxid-Partikeln. Daher wurde eine gemeinsame Begründung für mikro- und nanoskaliges Cerdioxid erstellt. Mikro- und nanoskalige Cerdioxid-Partikel zeigen zudem sehr ähnliche aerodynamische Durchmesser, gelan­gen in die lungennahen Lymphknoten und führen konzentrationsabhängig zu einer Entzündung in der Lunge (Gosens et al. 2014).

In einer Langzeit-Inhalationsstudie mit nanoskaligen Cerdioxid-Partikeln (NM-212) wurden eindeutig Cerdioxid-Agglomerate und Cerphosphat im Knochengewebe von Ratten gefunden (Tentschert et al. 2020). Die von Tentschert et al. (2020) verwendeten Nachweismethoden (ICP-MS, ToF-SIMS) sind in der Lage, sowohl Cerdioxid als auch Cerphosphat im festen Zustand nachzuweisen. Die Argumentation von Tentschert et al. (2020) ist stringent formuliert und nachvollziehbar, dennoch ist nicht eindeutig geklärt, in welcher Form Cerdioxid in die Knochen gelangt und dort als Cerphosphat eingebaut wird. Möglicherweise gelangen lösliche Anteile des Cerdioxids oder Cerdioxid-Nanopartikel über das Knochenmark in die Knochen und mineralisieren als Cerphosphat in Calciumphosphat-haltigen Bereichen. Eine weitere Möglichkeit ist, dass aktive Transportmechanismen ähnlich wie beim Bariumsulfat zum Tragen kommen.

Cerdioxid-Partikel wirken im biologischen System vorrangig aufgrund ihres Redoxpotentials an der Partikeloberfläche, jedoch kann es sich bei dem toxischen Agens auch um lösliche Anteile des Cerdioxids handeln. Grundsätzlich kann die Löslichkeit in einem biologischen System, ähnlich wie bei Bariumsulfat, höher sein als in Wasser. Die von Tentschert et al. (2020) beschriebene Makrophagen-Überladung bei einer Konzentration von 3 mg/m^3^ ist eher auf den toxischen Einfluss der gelösten Cerdioxid-Anteile auf die Makrophagen zurückzuführen. Dieser Effekt auf die Makrophagen könnte auch der bei Schwotzer et al. (2017) beschriebenen reduzierten Clearance bei 3 mg/m^3^ und der leicht reduzierten Clearance bei 1 mg/m^3^ zugrunde liegen. 

In der Langzeit-Studie von Ernst et al. (2018) wird in der histopathologischen Untersuchung eine Infiltration mit Entzündungszellen aus hauptsächlich mononukleären Zellen und wenigen Granulozyten beobachtet. Cerdioxid indu­ziert eine spezifische granulomatöse Entzündung in der Lunge, bestehend aus Mikrogranulomen und Riesenzellen (Synzytien). Viele dieser Mikrogranulome stellen sich als Immungranulome mit einem Lymphozytensaum dar (Ernst et al. 2018). Bei einer hohen Belastung („overload“) mit einem granulären biobeständigen Staub kommt es zu einem Zerfall von Makrophagen und einer Wieder-Freisetzung von Partikeln. Hierdurch würde in einer (sub)chronischen Studie eine Reaktion entsprechend der akuten Phase angestoßen, d. h. es wäre ein Anstieg bzw. eine Akkumulation von PMNs zu erwarten (Hartwig 2012).

In einer 90-Tage-Studie lag die ermittelte NOEC für mikroskaliges Cerdioxid bei < 5 mg/m^3^ (Bio-Research Laboratories Ltd 1994; US EPA 2009). In Studien mit nanoskaligen Cerdioxid-Partikeln zeigte sich eine NOAEC von < 1 mg/m^3^ (Schwotzer et al. 2017). In einer umfangreichen 2-Jahre-Inhalationsstudie mit nanoskaligen Cerdioxid-Partikeln an Ratten riefen bereits Expositionskonzentrationen ab 0,1 mg/m^3^ eine statistisch signifikante granulomatöse Ent­zündung sowie eine interstitielle Fibrose der Lunge hervor. Insgesamt unterlag der Schweregrad und die Inzidenz der Befunde in allen Gruppen einer interindividuellen Variabilität, insbesondere in den niedrigsten Kon­zentrationsgruppen war die Variabilität des Schweregrades jedoch besonders deutlich ausgeprägt (Ernst et al. 2018; Schaudien et al. 2018).

Ausgehend von den Ergebnissen der Langzeitstudie von Ernst et al. (2018) wird die Konzentration von 0,1 mg Cer­dioxid/m^3^ als LOAEC angesehen. Für die Ableitung des MAK-Wertes wurde das HEC-Verfahren angewendet (Parameter wie in Hartwig 2012). Die humanäquivalente Konzentration berechnet sich dabei nach folgender Formel:

HEC = C_T_ × (AgV_T_/AgV_H_) × (NF_H_/NF_T_) × (ELR_H_/ELR_T_) × (DF_T_/DF_H_)

Dabei entsprechen C_T_ der Expositionskonzentration im Tierversuch, AgV dem gewichteten täglichen Atemvolumen, ELR der durchschnittlichen Eliminationsrate und NF einem Normalisierungsfaktor, wobei hier die Lungenoberfläche von Ratte und Mensch als Bezugsgröße angenommen wurde. DF entspricht den deponierten Fraktionen im tracheobronchialen und alveolären Bereich. Zur Berechnung dieser Fraktionen wurde das Programm Multiple-Path Particle Dosimetry Model (MPPD; Version 3.04) verwendet. 

Als Eingangsparameter dienten neben der LOAEC von 0,1 mg/m^3^ ein MMAD von 1,4 µm, eine GSD von 2,3 sowie eine Agglomerat-Dichte von 2 g/cm^3^ (Keller et al. 2014). Unter Berücksichtigung der Ganzkörperexposition im Tierversuch und einem erhöhten Atemvolumen beim Arbeitnehmer ergibt sich im tracheobronchialen und alveolären Bereich für die Ratte eine deponierte Fraktion von 0,0595 und für den Menschen von 0,129 (Tabelle 20).

**Tab.20:** Deponierte Fraktion von Cerdioxid bei der Ratte und beim Menschen nach MPPD Version 3.04

MPPD; Version 3.04	**Mensch**	**Ratte**
**Spezies/Modell Info**		
Spezies/Geometrie	Human limited	Long Evans Rat asymmetric
FRC	3300 ml	4 ml
Head Volume	50 ml	0,42 ml
Atemweg (Breathing Route)	Normal forciert	Nasenatmung
**Atemparameter**		
Atemzugvolumen	1040 ml	2,1 ml
Atemfrequenz	20/Minute	102/Minute
Inspirationsfraktion	0,5	0,5
**Berechnete Depositionsfraktion**		
Kopfbereich	0,4785	0,4266
tracheobronchial	0,0354	0,0149
alveolär	0,0936	0,0446
tracheobronchial + alveolär	0,129	0,0595

FRC: funktionelle Residualkapazität

Unter Berücksichtigung der ermittelten Werte ergibt sich die folgende humanäquivalente Konzentration:

HEC = 100 µg/m^3^ × 0,00837 × 192,7 × 0,15 × (0,0595/0,129) = 11,2 µg/m^3^

Entsprechend der Vorgehensweise der Kommission (siehe Abschnitt I der MAK- und BAT-Werte-Liste; DFG 2023) wird unter Berücksichtigung der Extrapolation von der LOAEC auf eine NAEC (1:3) und der Berücksichtigung des für die Übertragung von Tierversuchen auf den Menschen verwendeten Faktors (1:2) ein** MAK-Wert von 2 µg/m^3^** für die alveolengängige Fraktion abgeleitet.

**Spitzenbegrenzung. **Der kritische Effekt von Cerdioxid ist die systemische Wirkung auf die Lunge. Die alveolengängige Fraktion wird daher der Spitzenbegrenzungs-Kategorie II zugeordnet. Bei einer Expositionskonzentration von 0,5 mg/m^3^ beträgt die Halbwertszeit in der Rattenlunge 40 Tage (960 Stunden), wobei der primäre Clearance-Mechanismus bei dieser Konzentration die Elimination über die Makrophagen ist (Keller et al. 2014). Bei der geringen Löslichkeit von Cerdioxid ist es unwahrscheinlich, dass die Halbwertszeit bei geringeren Konzentrationen wesentlich kürzer ist. Zudem wird nicht erwartet, dass die Redoxaktivität von Cerdioxid bei kurzzeitiger Konzentrationserhöhung überproportional zunimmt. Der Überschreitungsfaktor wird daher auf 8 festgelegt. Cerdioxid ist im Draize-Test am Auge minimal reizend (ECHA 2022). Eine lokale Irritation oder eine akute Reizwirkung im Atemtrakt ist daher bei den zulässigen 16 µg/m^3^ nicht zu erwarten. 

**Krebserzeugende Wirkung. **Aus der vorliegenden Langzeitstudie von Ernst et al. (2018) ergibt sich kein Hinweis auf eine kanzerogene Wirkung von Cerdioxid-Nanopartikeln. In Bezug auf Neoplasien wurden nach einer inhalativen Exposition gegen Cerdioxid-Nanopartikel über 24 Monate bzw. über 24 Monate plus sechs Monaten Erholungszeit keine statistisch signifikanten Unterschiede zur Kontrollgruppe ermittelt. Die in dieser Studie verwendete Nachweistechnik mit über 60 Schnitten pro Lunge verfügt laut Untersuchungsergebnissen von Kolling et al. (2008) über eine zwei- bis dreifach höhere Detektionsrate im Vergleich zu den standardmäßig verwendeten Untersuchungsmethoden.

**Keimzellmutagene Wirkung. **In Fütterungsstudien an Ratten traten Mikronuklei nach Gabe von 300 mg nano­skaligem Cerdioxid/kg KG und Tag, jedoch nicht nach Gabe von 30 mg/kg KG und Tag auf. Zur toxikokinetischen Übertragung des NOAEL von 30 mg/kg KG und Tag in eine Konzentration in der Luft am Arbeitsplatz werden berück­sichtigt: eine orale Resorption von 0,1 %, eine inhalative Aufnahme von 1 % (Abschnitt 3.1.1), der dem toxikokinetischen Unterschied zwischen der Ratte und dem Menschen entsprechende speziesspezifische Korrekturwert (1:4), das Körper­gewicht (70 kg) und das Atemvolumen (10 m^3^) des Menschen. Damit errechnet sich eine Konzentration von 5,26 mg/m^3^. Aufgrund des großen Abstandes zum MAK-Wert wird die Wirkstärke als so gering erachtet, dass bei Einhaltung des MAK-Wertes nur ein geringer Beitrag zum genetischen Risiko zu erwarten ist. Cerdioxid wird daher in die Kategorie 5 für Keimzellmutagene eingestuft.

**Fruchtschädigende Wirkung. **Beim Menschen liegen keine belastbaren Daten vor. 

Eine pränatale Entwicklungstoxizitätsstudie nach gültiger OECD-Prüfrichtlinie 414, welche die gesamte Organogenese umfasst und die komplette Untersuchung auf Teratogenität beinhaltet, liegt nicht vor. In zwei kombinierten, gemäß der OECD-Prüfrichtlinie 422 durchgeführten Toxizitätsstudien inklusive Screening-Test auf Reproduktions-/Entwicklungstoxizität, kommt es nach wiederholter Verabreichung mittels Schlundsonde an Sprague-Dawley-Ratten bis zur eingesetzten Höchstdosis von 1000 mg Cerdioxid-NP/kg KG und Tag zu keinen Effekten an Muttertieren oder Nachkommen (CiT Safety & Health Research Laboratories 2010; Lee et al. 2020).

Eine mechanistische Studie weist auf eine Beeinträchtigung der Alveolenbildung in der Lunge und auf ein verringertes Gewicht bei den Nachkommen von C57BL6/J-Mäusen nach dreimaliger intratrachealer Applikation von 0,1 mg CeO_2_-NP/Tier (die Autoren errechnen ohne nähere Angaben eine äquivalente inhalierte Konzentration von 2,8 mg/m^3^ für eine Exposition von acht Stunden pro Tag und fünf Tagen) am 2,5.; 9,5. und 16,5. Trächtigkeitstag bei gleichzeitiger Maternaltoxizität an der Lunge hin (Paul et al. 2017). Da nur eine Dosis getestet wurde, ist eine Angabe einer Dosis-Wirkungs-Beziehung nicht möglich. Eine Tabelle mit einer Übersicht zu den Reproduktionsdaten fehlt. Unklar bleiben die Anzahl und das Geschlecht der untersuchten Tiere sowie die Anzahl untersuchter Plazenten bzw. Lungen. Zur Klärung sind weitere Studien statt mit intratrachealer Bolusgabe, für die wenig Relevanz für den Arbeitsplatz gegeben ist, mit inhalativer Gabe und Untersuchung der Lunge notwendig. 

Da keine pränatale Entwicklungstoxizitätsstudie nach gültiger OECD-Prüfrichtlinie 414 vorliegt, welche die ­gesamte Organogenese umfasst und die komplette Untersuchung auf Teratogenität beinhaltet, wird Cerdioxid der Schwanger­schaftsgruppe D zugeordnet.

**Hautresorption. **Cerdioxid gehört zu den schwer löslichen und sehr stabilen Cer-Verbindungen. Eine Diffusions­kammer-Studie zur transdermalen Resorption von nanoskaligem Cerdioxid zeigt eine geringe Permeation in die intakte Epidermis hinein, jedoch keine erhöhten Konzentrationen in der Rezeptorflüssigkeit gegenüber Kontrollproben ohne Nanopartikel. Klinische Symptome nach dermaler Applikation von Cerdioxid wurden im Tierversuch nicht beobachtet. Demnach ist die Hautresorption von Cerdioxid vernachlässigbar und Cerdioxid wird nicht mit „H“ markiert.

**Sensibilisierende Wirkung. **Trotz weitverbreiteter Verwendung von Cerdioxid als Schleifmittel liegen bis auf einen negativen Befund keine Daten zur kontaktsensibilisierenden Wirkung von Cerdioxid vor. Weder tierexperimentelle noch In-vitro-Untersuchungen geben einen Hinweis auf eine hautsensibilisierende Wirkung von mikro- und nanoskaligem Cerdioxid. Insgesamt wird die dermale Aufnahme von Cerdioxid bei intakter Haut als gering erachtet. Es erfolgt daher keine Markierung mit „Sh“.

Es liegen keine Hinweise auf eine atemwegssensibilisierende Wirkung von mikroskaligem Cerdioxid vor. Für nanoskaliges Cerdioxid liegen keine Hinweise auf eine eigenständige atemwegssensibilisierende Wirkung vor. Es erfolgt daher keine Markierung mit „Sa“.
